# The evolutionary heritage and ecological uniqueness of Scots pine in the Caucasus ecoregion is at risk of climate changes

**DOI:** 10.1038/s41598-021-02098-1

**Published:** 2021-11-24

**Authors:** M. Dering, M. Baranowska, B. Beridze, I. J. Chybicki, I. Danelia, G. Iszkuło, G. Kvartskhava, P. Kosiński, G. Rączka, P. A. Thomas, D. Tomaszewski, Ł. Walas, K. Sękiewicz

**Affiliations:** 1 Institute of Dendrology, Polish Academy of Sciences, Parkowa 5, 62-025 Kórnik, Poland; 2 Poznań University of Life Sciences, Wojska Polskiego 71a, 60-625 Poznań, Poland; 3 Department of Genetics, Faculty of Biological Sciences, Kazimierz Wielki University, Powstańców Wielkopolskich 10, 85-090 Bydgoszcz, Poland; 4 Faculty of Agricultural Science and Bio-System Engineering, Georgian Technical University, Guramishvili Str. 17, 0192 Tbilisi, Georgia; 5 Faculty of Biological Sciences, University of Zielona Góra, Prof. Z. Szafrana 1, 65-516 Zielona Góra, Poland; 6 School of Biological Sciences, Keele University, Staffordshire, ST5 5BG UK

**Keywords:** Evolutionary biology, Population genetics

## Abstract

Scots pine is one of the most widely occurring pines, but future projections suggest a large reduction in its range, mostly at the southern European limits. A significant part of its range is located in the Caucasus, a global hot-spot of diversity. Pine forests are an important reservoir of biodiversity and endemism in this region. We explored demographic and biogeographical processes that shaped the genetic diversity of Scots pine in the Caucasus ecoregion and its probable future distribution under different climate scenarios. We found that the high genetic variability of the Caucasian populations mirrors a complex glacial and postglacial history that had a unique evolutionary trajectory compared to the main range in Europe. Scots pine currently grows under a broad spectrum of climatic conditions in the Caucasus, which implies high adaptive potential in the past. However, the current genetic resources of Scots pine are under high pressure from climate change. From our predictions, over 90% of the current distribution of Scots pine may be lost in this century. By threatening the stability of the forest ecosystems, this would dramatically affect the biodiversity of the Caucasus hot-spot.

## Introduction

Since the beginning of the industrial era we have lost 32% of the world’s forest, and over 8,000 of the world’s 60,065 tree species are threatened with extinction^1^. However, the genetic components of our trees should be a conservation priority as well^2^^,^^3^. Three perspectives of biodiversity that mirror the evolutionary legacy of the species are genetic diversity (neutral and adaptive DNA polymorphisms), phylogeographic diversity (intraspecific evolutionary lineages) and phylogenetic diversity (intraspecies taxonomic units, e.g. subspecies or varieties). All these facets of genetic biodiversity should be more explicitly embodied in the conservation priorities that have been recently articulated by the scientific community^2,4^.

Scots pine (*Pinus sylvestris* L.) is the most widely distributed pine species, yet its occurrence is seriously threatened by climate change, especially in the isolated and relic populations at the southern fringes of its range^5,6^. In the South Caucasus and Asia Minor Scots pine is a Tertiary relic with a patchy distribution in mountainous areas separated from the current main boreal range by a distributional gap of over 1,000 km. By the end of 2080, approximately one-third of the current distribution of Scots pine in Europe might be lost, with southern stands being most affected^5,6^. If this decline happens, it will greatly impoverish the genetic, phylogeographic and phylogenetic diversity of Scots pine, that may ultimately affect adaptive potential of the species.

In a majority of organisms, genetic diversity is unevenly distributed within the range of a species. The 'central-marginal’, 'southern richness and northern purity' or 'leading-edge *vs*. rear-edge' hypotheses exemplify the attempts to conceptualize the observed spatial organization and determinants of genetic diversity by drawing attention to species’ ecology and evolution^7–9^. All these models predict a distinct genetic composition of peripheral populations, which evolve to be characterized by either a low genetic diversity and adaptability or unique DNA polymorphisms that might be crucial/valuable in future adaptation^10,11^. Therefore, peripheral populations have become significant in discussions of adapting to climate change^12^. Studies indicated that peripheral populations may have both high neutral^13^ and adaptive genetic variation^11^. In this light, the isolated populations of tree species at the southern edge of their geographic range, such as those of Scots pine, are of particular interest^8,14,15^.

Locations of glacial refugia, colonization pathways and intraspecific divergence patterns are important because they can give insight into the possible genetic consequences of a range shift in the context of climate change. The Caucasus has regularly been highlighted as a potential Last Glacial Maximum (LGM) refugial area for different organisms^16–18^ including Scots pine^19^. However, neither the exact number and location of the refugia, nor their contribution to recolonization and genetic structure, have been identified. The palaeoclimatic models and pollen data suggest that the major refugia for plants within the Caucasus were in the Colchis (a lowland on the eastern Black Sea coast), the Pontic Mts. in Turkey and the Hyrcanian Forest in the Alborz Mts. of Iran^16,20^. Numerous studies have documented how different factors have shaped the distribution and the genetic patterns of Scots pine in different part of the range, including Asia Minor^21–29^. However, none of these studies included a comprehensive representation of populations from the South Caucasus (Georgia), despite this region representing the largest part of the species range in the Caucasus ecoregion. The most recent paper relevant for the region focused on populations located in the Northern Caucasus (Russia)^30^. Based on cytoplasmic markers, Semerikov et al.^30^ suggested a probable time of the divergence between European and the Caucasian lineages at ca. 1 Mya but only partly reconstructed the possible migration routes in the region due to insufficient sampling.

In this study, we applied species distribution models (SDMs) and the analysis of the mitochondrial DNA sequences (mtDNA) and nuclear microsatellite markers (nSSRs) in *P. sylvestris* var. *hamata* Steven from Asia Minor and the South Caucasus to obtain insight into the key demographic and biogeographical processes (local persistence, range shifts, divergence time, genetic bottlenecks) governing the species evolutionary history in the region. Specifically, we asked several questions: (1) Did Scots pine in Asia Minor and the Caucasus survive the last glacial period in a single refugium or in multiple refugia and where were they located? (2) Is the present genetic diversity geographically structured? (3) Does the structure reflect the vicariance process in isolated refugia? (4) What theoretical model describes best the spatial patterns of genetic diversity and differentiation? (5) Does geographical marginality represent the climatic marginality of Scots pine in the Caucasus ecoregion? (6) What are the possible changes in species distribution in the Caucasus ecoregion according to different climatic scenarios?

## Materials and methods

### Sampling and genotyping

**Figure 1:**
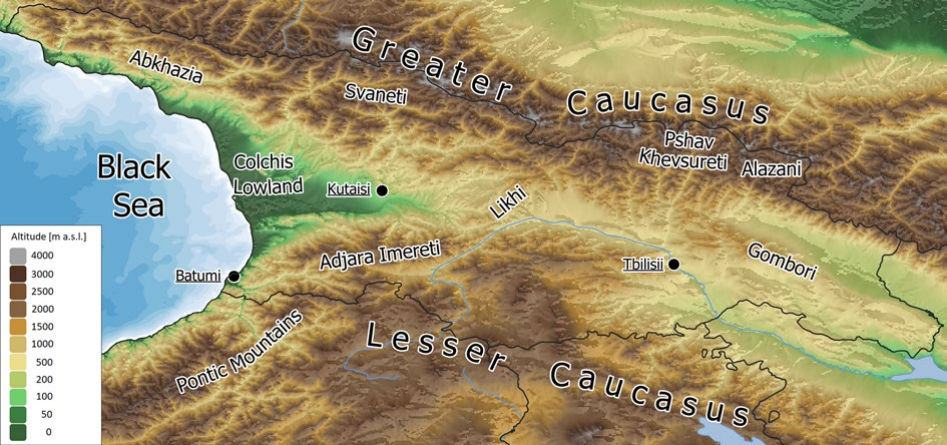
The major geographic  regions of the Caucasus ecoregion. Map generated with QGIS 3.16 (https://qgis.org/en/site/).

**Figure 2:**
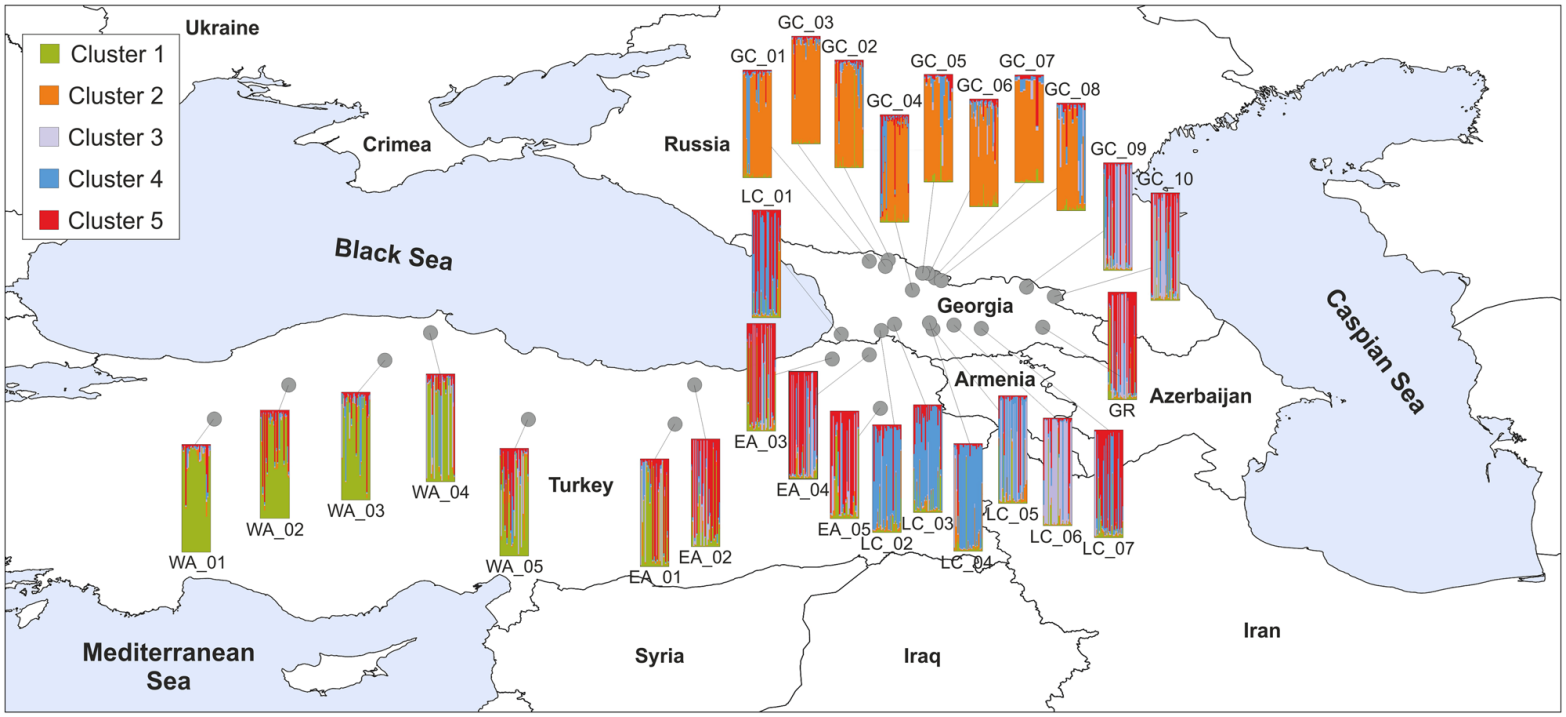
Location of the studied populations of Scots pine in the South Caucasus and Anatolia and the results of the STRUCTURE analysis conducted using nuclear microsatellites. Bar plots present the proportion of membership of each individual in the five clusters indicated (K = 5). Map generated with QGIS 3.16 (https://qgis.org/en/site/).

Needles were collected from 28 populations (804 individuals) in the natural species range covering Georgia (18 populations) and Turkey (10 populations). More specifically, we sampled seven populations in the Lesser Caucasus (LC), 10 populations in the Greater Caucasus (GC), a single population in the Gombori range that links the Greater Caucasus and the Lesser Caucasus (GR), five populations in West Anatolia (WA), and five populations in East Anatolia (EA) (Figs. 1, 2, Supplementary file 1, Table S1). Genomic DNA was extracted using the CTAB protocol^31^.

We used two mtDNA regions *nad7* and *nad1* which have proved to be polymorphic in Scots pine and interpretable in terms of glacial refugia^23,26^. PCR reactions were performed according to Dering et al.^26^. The results of genetic analysis conducted in the Caucasus were compared with mtDNA data presented in Dering et al.^26^ which covered the European and Asiatic range of the species with a single population located in the Caucasus.

Thirteen nuclear microsatellite markers (nSSR) were selected for the study^32,33^ and combined in three multiplex PCRs: Multiplex I—Psyl57, Psyl25, Psyl17, Psyl36,Multiplex II—Psyl44, Psyl42, Psyl19, Psyl2, Psyl16 and Multiplex III—SPAC7.14 SPAC11.4 SPAC11.8 SPAC12.5. Details of the reactions’ conditions are presented in Supplementary file 1 (Table S2). PCR products were analysed using a capillary genetic analyzer ABI PRISM 3130XL with internal size standard GeneScan 500LIZ (Applied Biosystems, Foster, California). Genotypes were scored using the GeneMapper v. 4.0 software (Applied Biosystems).

### Genetic data analysis

#### *Diversity and differentiation*

GenALEx v. 6.5^34^ was used to determine the mean number of alleles (*A*) and the number of private alleles (*P*_*A*_). Observed (*H*_*O*_) and expected (*H*_*E*_) heterozygosity per population were calculated with INEST v. 2.0^35^, whereas allelic richness (*AR*) was computed using FSTAT v. 2.9.3^36^, and null alleles (Null) in FreeNA^37^ (using the Dempster method). The difference among geographic regions (Greater Caucasus, Lesser Caucasus, Western Anatolia, and Eastern Anatolia) in mean allelic richness, gene diversity and *F*_*ST*_ was tested in FSTAT with a permutation test (10^3^ permutations). INEST was also used to estimate the inbreeding coefficient robust to null allele presence. The calculations were run using a total of 5 × 10^4^ MCMC iterations with every 200th updated and 5 × 10^3^ of burn-in. The Deviance Information Criterion (DIC) was used to choose the best model by comparing the inbred population model (*nfb* = null alleles, inbreeding coefficient and genotyping failures, *F*_*IS*_ > 0) with the random mating model (*nb* = null alleles, genotyping failures, *F*_*IS*_ = 0). The Excluding Null Alleles (ENA) correction implemented in the FreeNA software was used to adjust Wright's fixation index (*F*_*ST*_) for the null alleles presence. The significance of *F*_*ST*_ was tested using the bootstrapping method.

### Range-wide spatial genetic structure

The spatial genetic structure was examined in STRUCTURE v.2.3^38^. The analysis was based on the admixture model and correlated allele frequencies model. Ten independent runs for each *K* value were applied, ranging from 1 to 29 (i.e. the number of sampling locations + 1) and burn-in of 1 × 10^4^ steps, followed by 2 × 10^4^ MCMC iterations. Genotype clustering is a challenging task but finding the optimal number of clusters, called the K number, is an even more complicated issue, and there is no dominant approach in population genetics^39^. In this paper, we took advantage of an approach proposed by Puechmaille^40^ that has been shown to define K with a higher accuracy in comparison to commonly used ΔK^41^ or LnP(D)^38^. To do this, we used STRUCTURE SELECTOR^42^.

Due to the presence of natural barriers to gene flow in the study area, the genetic differentiation pattern may reflect population connectivity that is more a function of landscape features than a geographic distance. Therefore, we used the EEMS method (Estimated Effective Migration Surfaces) to investigate whether the observed population genetic structure is a function of migration rates that are shaped by the existing barriers and corridors^43^. The matrix of pairwise genetic dissimilarities was calculated over the triangular grid that covered the region of the South Caucasus and Anatolia (300 demes). The effective migration and diversity rates were estimated in five independent runs with 4 × 10^6^ MCMC iterations, 10^6^ of burin-in and thinning set to 9999. We checked for convergence and mixing, and visualized migration and diversity surfaces using the rEEMSplots package^43^. To provide a graphical summary of the observed genetic dissimilarities, the estimated rates were interpolated across geographical space. The effective migration and genetic diversity rates were presented on a log10 scale, hence log(*m*) = 1 means that the effective migration is tenfold faster than the average.

### Demographic history

To test whether the studied populations reveal signs of a genetic bottleneck, the *M*-ratio method implemented in INEST was used. The critical *M*-value (the Garza-Williamson index^44)^ was estimated by simulating a demographically-stable population (using the standard coalescent simulations) under the TPM model (two-phased mutation model) assuming a proportion of one-step mutations (*ps*) of 0.22 and mean size of multi-step mutations (*Δg*) of 3.1. The equilibrium *M*-value was estimated as a mean across 10^4^ coalescent replicates. The Wilcoxon’s signed-rank test (markers treated as independent replicates) was applied to test for significant difference between the observed and the equilibrium *M*-value.

Demographic scenarios of the Scots pine populations were tested through the Approximate Bayesian Computation in DIYABC 2.1.0^45^. The investigated populations were assembled into five groups revealed by STRUCTURE (see Results): West Greater Caucasus (GC_01-GC_08), West Lesser Caucasus (LC_01-LC_05), East Caucasus (GC_09, GC_10, LC_06-LC_07 and GR), West Anatolia (WA_01-WA_05, EA_01) and East Anatolia (EA_02-EA_05). Four scenarios were focused on divergence events (Scenarios 2–5), and one on an admixture (Scenario 1) (Supporting file 1, Fig. S1). These scenarios were built based on suggestions about the location of refugial areas in the Caucasus, results of population pairwise _ST_ and STRUCTURE clustering. In the initial analysis, it was difficult to satisfactorily discriminate between scenarios according to their fit to the observed data. Thus, we decided to split loci into two groups based on their polymorphism. The details of the DIYABC procedure are given in the Supplementary file (Supplementary file 1, Table S3). We performed 7 × 10^5^ coalescent simulations for five tested scenarios. At first, scenario-prior combinations were evaluated using PCA to identify the correspondence between priors and observed data. The competing scenarios were compared by calculating their posterior probabilities using a logistic regression on the 1% of simulated data closest to the observed data. The confidence in scenario choice was evaluated by generating 1,000 data sets from priors and by computing Type I and Type II errors. The posterior distributions of the genetic and demographic parameters were estimated using a local linear regression approach on the 1% of the simulated data closest to our observed data set, after logit transformation of the parameters. In order to convert the time of divergence presented by DIYABC as a number of generations into calendar years we assumed a generation time for Scots pine to be 20–25 years, similar to other authors^46^. This value reflects the age in which the species enter the reproductive phase. In contrast, 100 years used by^30,47^ reflects more the average species life-span than the generation time.

### Ecological niche modelling

The theoretical range of *P. sylvestris* in the Caucasus region was estimated in MAXENT 3.3.2^48^. A dataset of 134 occurrence points (one point per grid cell of 1 × 1 km) that included published locations, the GBIF (14 November 2019, GBIF Occurrence Download https://doi.org/10.15468/dl.rcksos), and our data from the Caucasus, was used for SDM (Supplementary file 1, Table S1). All points used were manually validated. A set of 19 bioclimatic variables, downloaded from CHELSA database for current conditions and for CCSM4 Last Maximum Glaciation model^49,50^ were employed with a 30 arc-sec resolution. Additionally, we added a raster layer of soil data based on the World Reference Base soil classification (TAXNWBR) with a resolution of 250 m from the SoilGrids^51^. The correlation between climatic variables was evaluated with the *raster.cor* function from the ENMTools package in R 3.4.3^52,53^. To reduce collinearity between variables, *bio11*, *bio14*, and *bio17* were excluded from further analyses. Four analyses were performed: 1) for LGM and 2) for the current conditions with and 3) ignoring the soil raster, and 4) for the future according to three Representative Concentration Pathways (RCPs): RPC 2.6 (an increase of average temperature by 1 °C before the year 2100), RCP 4.5 (+ 1.8 °C before the year 2100), and RCP 8.5 (+ 3.7 °C before the year 2065)^54^. The bootstrap procedure with 100 replicates with a 'random seed' option was used, 20% of data were used as test points for model evaluation. Output was set to a logistic, convergence threshold to 0.00001 and maximum iterations were set to 10,000. Area Under the Curve (AUC) was used to evaluate the final model performance^55^. Results were visualized in QGIS 3.4.8 'Madeira' (QGIS Development Team, 2012). Suitable area and average altitude in the theoretical range of species were calculated in SAGA GIS software^56^.

To investigate the climatic distinction of the Caucasian and Anatolian stands of Scots pine in reference to the whole natural range, we plotted current annual average climate variables for these areas, retrieved from CHELSA (period 1979–2013). In this analysis, we focused on the driest and hottest parts of the Caucasus ecoregion, so the North Caucasus (the Russian part) was excluded. We used the variables that gave the highest contribution for the South Caucasus and Anatolia in the analysis of the current and the predicted LGM distribution. Since precipitation and temperature are the two major limiting factors of plant growth and reproduction^57^, and are also predicted to change in adverse manner under climate changes, we used variables related to temperature and precipitation to better characterize the species niche in the region. We chose annual mean temperature (bio1) and annual precipitation (bio12) from CHELSA, and annualPET (annual potential evapotranspiration), aridityIndexThornthwaite (aridity index) and climaticMoistureIndex (relative wetness to aridity) from the ENVIREM database^58^. All raster layers used in the analysis were characterized by a 30 arc-sec resolution. The results were visualized as two-dimensional ecoplots presenting the Anatolian and Caucasian sites plotted against all remaining sites from the whole European natural range. The species' occurrence points for the analysis were downloaded from the chorological database provided by^59^ with a 30 arc-sec resolution. Additionally, we explored the species autecology by comparing several bioclimatic parameters (annual mean temperature, annual precipitation, mean temperature of the wettest quarter, the precipitation of the warmest quarter, aridity index, and potential evapotranspiration) among different distributional domains. Those geographical domains were defined as follows: the Alps, the Balkans, Central Europe, the Pyrenees, East Europe, Scandinavia, Scotland, the Caucasus and Anatolia (Supplementary file 1, Fig. S2). Tukey's test was used to test for significant differences in the average values of the parameters tested.

Additionally, the environmental differences between the four major geographic subregions determined by climatic differences in which the species was sampled were verified by PCA. These subregions were West Anatolia, East Anatolia, the Greater Caucasus and the Lesser Caucasus. The analysis was performed on 28 populations using the five bioclimatic variables that were most important in the MAXENT model (see Results). PCA was performed using the ‘*prcomp*’ function in R and visualised using the *ggbiplot* package in R package^52^.

## Results

### Genetic diversity and differentiation

All populations of Scots pine studied in this work were monotypic in terms of the mitochondrial markers used, and characterized by the mitotype *d* (Supplementary file 1, Fig. S3).

The highest average number of alleles was noted in population WA_02 from the West Anatolia (8.31) and lowest in GC_07 from the Greater Caucasus (5.23). The highest allelic richness (*AR*) was noted again in a population in the West Anatolia (WA_04, *AR* = 5.5) and the lowest in the population in the Gombori range (GR, *AR* = 4.2). The highest number of private alleles (*P*_*A*_ = 13) was present in populations from the Greater Caucasus, especially in those in the western part (Table 1). The West Anatolian populations were also rich in private alleles (*P*_*A*_ = 10). The low number of *P *_A_ characterized populations from the Lesser Caucasus—only three unique alleles were detected. The regional-level analysis for the presence of private alleles indicated that the highest P_A_ is in the Greater Caucasus (19), next in West Anatolia (15), East Anatolia (7) and the lowest number of regional *P *_A_is noted in the Lesser Caucasus. Heterozygosity was at a very similar level across all populations, but inbreeding varied greatly, ranging from 0.026 (GC_05) up to 0.280 (EA_03). In populations with a high *FIS* value, inbreeding was inferred as the likely cause of homozygosity excess (Table 1).

**Table 1:** Locations of natural populations of Scots pine investigated studied in this study along with summary of genetic variability estimated for each population across 13 nSSR loci (GC—the Greater Caucasus, LC—the Lesser Caucasus, GR—Gombori range, WA—West Anatolia, EA—East Anatolia).

Population ID	N	*A*	*AR*	*P*_*A*_	*H*_*O*_	*H*_*E*_	*F*_*ISNull*_	*Null*
GC_01	33	7.46	5.22	4	0.478	0.597	0.036	0.087
GC_02	30	6.61	4.89	2	0.445	0.595	0.033	0.103
GC_03	31	6.77	4.78	1	0.447	0.568	0.034	0.090
GC_04	30	7.31	5.27	1	0.521	0.601	0.064^inbr^	0.040
GC_05	18	5.84	4.90	1	0.509	0.609	0.026	0.074
GC_06	30	6.69	4.87	4	0.501	0.564	0.035^inbr^	0.046
GC_07	11	5.23	5.14	0	0.531	0.614	0.031	0.060
GC_08	19	5.92	4.97	0	0.451	0.586	0.097	0.108
GC_09	33	6.46	4.81	0	0.424	0.585	0.071^inbr^	0.092
GC_10	32	6.69	4.94	0	0.381	0.591	0.267^inbr^	0.043
GR	31	5.46	4.21	0	0.433	0.564	0.036^inbr^	0.101
**Average/total**		**6.40**	**4.91**	**13**	**0.465**	**0.588**	**0.066**	**0.077**
LC_01	30	6.31	4.59	1	0.451	0.541	0.064^inbr^	0.060
LC_02	30	6.08	4.80	1	0.472	0.540	0.057^inbr^	0.099
LC_03	30	6.92	5.12	1	0.518	0.550	0.022	0.036
LC_04	31	6.38	4.87	0	0.442	0.560	0.117^inbr^	0.042
LC_05	32	7.08	5.03	0	0.428	0.624	0.090	0.127
LC_06	30	6.23	4.78	0	0.399	0.568	0.077	0.122
LC_07	31	6.61	4.76	0	0.444	0.531	0.076	0.076
**Average/total**		**6.51**	**4.85**	**3**	**0.450**	**0.559**	**0.072**	**0.080**
WA_01	25	7.23	5.19	2	0.466	0.566	0.135^inbr^	0.036
WA_02	31	8.31	5.42	3	0.473	0.573	0.165^inbr^	0.013
WA_03	30	7.15	5.22	0	0.493	0.608	0.120	0.090
WA_04	30	7.85	5.47	2	0.422	0.595	0.254^inbr^	0.016
WA_05	30	7.54	5.23	3	0.456	0.594	0.108^inbr^	0.063
**Average/total**		**7.62**	**5.31**	**10**	**0.462**	**0.587**	**0.156**	**0.044**
EA_01	31	7.77	5.42	3	0.407	0.598	0.114^inbr^	0.119
EA_02	28	7.08	5.09	0	0.378	0.571	0.258^inbr^	0.034
EA_03	30	6.92	5.07	0	0.385	0.584	0.280^inbr^	0.025
EA_04	34	7.31	5.19	1	0.429	0.597	0.131^inbr^	0.058
EA_05	24	6.77	5.29	0	0.436	0.584	0.089^inbr^	0.078
**Average/total**		**7.17**	**5.21**	**4**	**0.407**	**0.587**	**0.174**	**0.063**

Bold values show average values of the parameters for regions.

N—number of analysed individuals; *A*—average number of alleles; *AR*_(10)_—allelic richness based on minimum sample size; *P*_*A*_—number of private alleles; *H*_*O*_—observed heterozygosity; *H*_*E*_—expected heterozygosity; *F*_*IS*_—inbreeding coefficient estimated including ‘null alleles’ correction; *Null*—null allele frequency.

### Range-wide spatial genetic structure

The most optimal number of clusters was K = 5 (Fig. 2, Supplementary file 1, Fig. S3, Table S4). The Anatolian populations were split into two groups, roughly in line with their east–west distribution in the Pontic Mts. Cluster I contained all the West Anatolian populations and a single East Anatolian population (WA_01-05 and EA_01), while Cluster V contained four East Anatolian (EA_02-05) and highly admixed populations of LC_07 and GR. The Cluster IV grouped the populations from the West Lesser Caucasus (LC_01-04) and populations GC_01-08 from the West Greater Caucasus formed Cluster II. By contrast, populations LC_05-06 from the Lesser Caucasus and GC_09-10 from the Greater Caucasus were highly admixed, and their common feature was the presence of the specific gene pool denoted as Cluster III. However, the highest value of the coefficient of membership (Q) to this cluster was noted only in population LC_06 (71%) while in the remaining populations coefficient Q was much lower, ranging from 31 to 50% (Supplementary file 1, Table S4).

**Figure 3:**
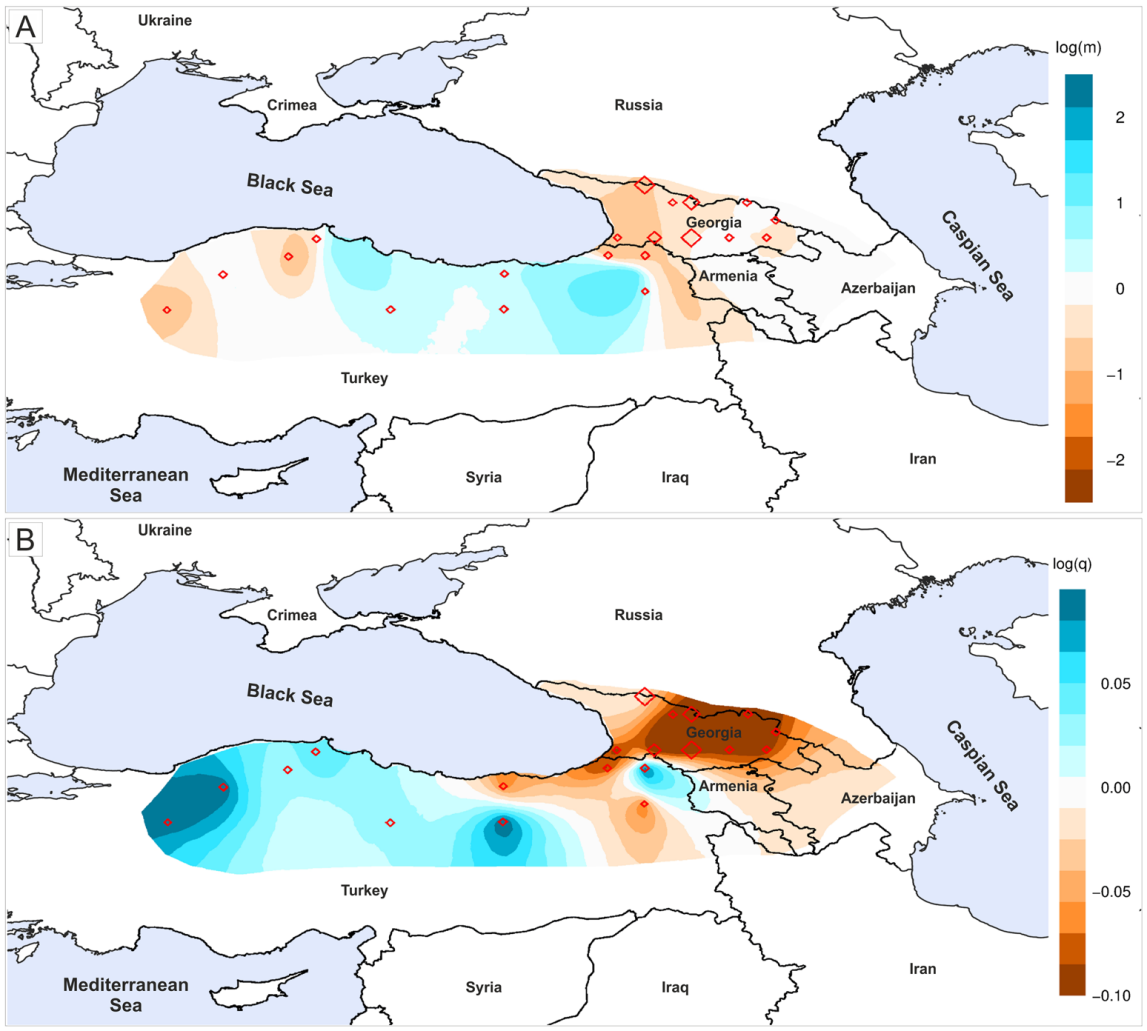
Estimated Effective Migration Surfaces analysis performed for 28 populations of Scots pine in the South Caucasus and the Anatolia based on nuclear microsatellites. (**A**) migration rates among populations, (**B**) diversity rates. The results are presented on a log-scale. Map generated with QGIS 3.16 (https://qgis.org/en/site/).

The MCMC chain convergence test in the EEMS analysis confirmed the estimation reliability (Supplementary file 1, Fig. S5). The results indicated less gene flow than expected in the western part of the Caucasus (Fig. 3). The zone of low genetic connectivity runs from the Colchis Plain along with neighbouring high mountain areas that include the Adjara Imereti range up to Svaneti range (Fig. 1, 3A). In the East Caucasus, dissimilarity among populations and thus gene flow followed the expectation of IBD. However, inferred gene flow was higher than expected in East Anatolia, and lower in West Anatolia. Estimates of the effective diversity clearly pinpointed Anatolia as the centre of Scots pine genetic diversity as individuals from that region were genetically more dissimilar than expected, except for three populations from East Anatolia (Fig. 3B).

### Demographic history

Eight out of 28 populations showed significant signs of a genetic bottleneck (Supplementary file 1, Table S5), primarily in the Anatolian populations (WA_01, WA_02, WA_05, EA_01 and EA_04) with two populations from the Lesser Caucasus (LC_03 and LC_07) and a single population from the Greater Caucasus (GC_03).

**Figure 4:**
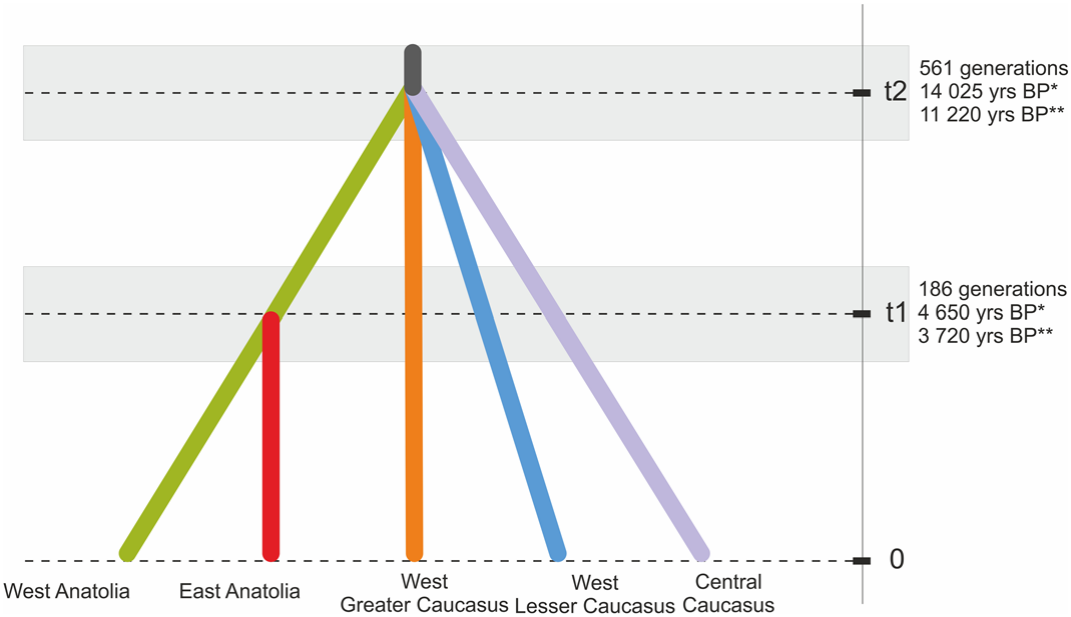
The best demographic scenario of divergence obtained for *Pinus sylvestris*  in the South Caucasus and Anatolia based on nuclear microsatellites using DIYABC. *25 years as the generation time; **20 years as the generation time. t1—time of the divergence between West and East Anatolian lineages; t2—time of the divergence from the most recent common ancestor; 0—current time.

Under the tested scenarios, the PCA-based analysis in DIYABC showed that the coalescent simulations constructed with the selected prior assumptions in Scenario 2 were best able to sufficiently reproduce the observed genetic data (Fig. 4, Supplementary file 1, Fig. S6 and Table S6) with the highest posterior probability of 0.8457 (95% CI 0.8025–0.8889, Supplementary file 1, Fig. S7). The proportion of incorrectly identified scenarios over 1000 test data sets for the logistic approach (the posterior predictive error) was 0.18. Type I error for Scenario 2 was 0.202, and the Type II error ranged from 0.016 to 0.084 (Supplementary file 1, Table S7). The PCA results for simulated data overlapped with the PCA results for the observed data, proving the reliability of our simulations (Supplementary file 1, Fig. S8).

Scenario 2 predicted a common origin for the Caucasian and Anatolian populations of Scots pine (Fig. 4). However, it also predicted that the Anatolian, West Greater Caucasus, West Lesser Caucasus and Central Caucasus populations diverged from the ancestral gene pool simultaneously. In contrast, the divergence of the West and East Anatolian populations took place later. The values of the original demographic and genetic parameters inferred in DIYABC are given in Table 2. The estimated divergence time back to the common ancestor was 561 generations ago (95% CI 147–1730 generations) while the split between the West Anatolian group and East Anatolian might have occurred 186 generations ago (95% CI 38.2–707 generations). The effective current population size of the West Anatolian group was the highest (N1 = 11,900), while the sizes of the remaining groups ranged from 3,650 for the West Lesser Caucasus (N5) to 6160 for the West Greater Caucasus (N2).

**Table 2:** Parameter estimates for the best demographic scenario (Scenario 2) indicated by DIYABC based on ABC method using nuclear microsatellites.

Parameter	Median	Q5%	Q95%
West Anatolia	1.19 × 10^4^	4.08 × 10^3^	2.70 × 10^4^
West Greater Caucasus	6.16 × 10^3^	2.02 × 10^3^	1.48 × 10^4^
Central Caucasus	4.58 × 10^3^	1.37 × 10^3^	9.07 × 10^3^
East Anatolia	4.98 × 10^3^	1.33 × 10^3^	9.21 × 10^3^
West Lesser Caucasus	3.65 × 10^3^	9.40 × 10^2^	8.61 × 10^3^
t1	1.86 × 10^2^	3.82 × 10	7.07 × 10^2^
t2	5.61 × 10^2^	1.47 × 10^2^	1.73 × 10^3^
µmic_1	5.04 × 10^–5^	1.08 × 10^–5^	9.32 × 10^–5^
pmic_1	1.44	2.05 × 10^–1^	5.77
µmic_2	6.51 × 10 ^-3^	9.39 × 10^–4^	1.00 × 10^–2^
pmic_2	3.73	9.48 × 10^–1^	4.91

### Ecological niche modelling

All MAXENT models showed a good fit with AUC greater than 0.97 (Table 3). The distribution of Scots pine in the Caucasus is mainly driven by water availability because the precipitation of the warmest quarter (*bio18*) has a relative contribution close to 50% in all models (Table 3). Soil type, with a relative contribution of 10.8%, also had a significant impact on distribution with haplic podzols being the most suitable. In the Caucasus, Scots pine is often found on cambisols (Supplementary file 1, Fig S9).

**Table 3:** Contribution of 19 environmental variables in the tested climate models.

	Model	Current with soil	LGM
	**AUC**	0.978	0.980
	*Variables:*		
*bio1*	Annual Mean Temperature	1.5	1.4
*bio2*	Mean Diurnal Range	0.8	0.7
*bio3*	Isothermality	0.9	1.4
*bio4*	Temperature Seasonality	2.6	4.0
***bio5***	**Max Temperature of Warmest Month**	**7.7**	**11.6**
*bio6*	Min Temperature of Coldest Month	3.7	5.0
*bio7*	Temperature Annual Range	0.7	0.4
***bio8***	**Mean Temperature of Wettest Quarter**	**10.0**	5.1
*bio9*	Mean Temperature of Driest Quarter	1.3	1.1
*bio10*	Mean Temperature of Warmest Quarter	1.6	1.7
*bio12*	Annual Precipitation	1.9	3.0
*bio13*	Precipitation of Wettest Month	0.9	0.9
*bio15*	Precipitation Seasonality	1.6	1.4
***bio16***	**Precipitation of Wettest Quarter**	**4.3**	**12.4**
***bio18***	**Precipitation of Warmest Quarter**	**48.7**	**48.9**
*bio19*	Precipitation of Coldest Quarter	1.0	1.0
	Soil Type	**10.8**	–

Bold values are those parameters that have the highest input into the model.

**Figure 5:**
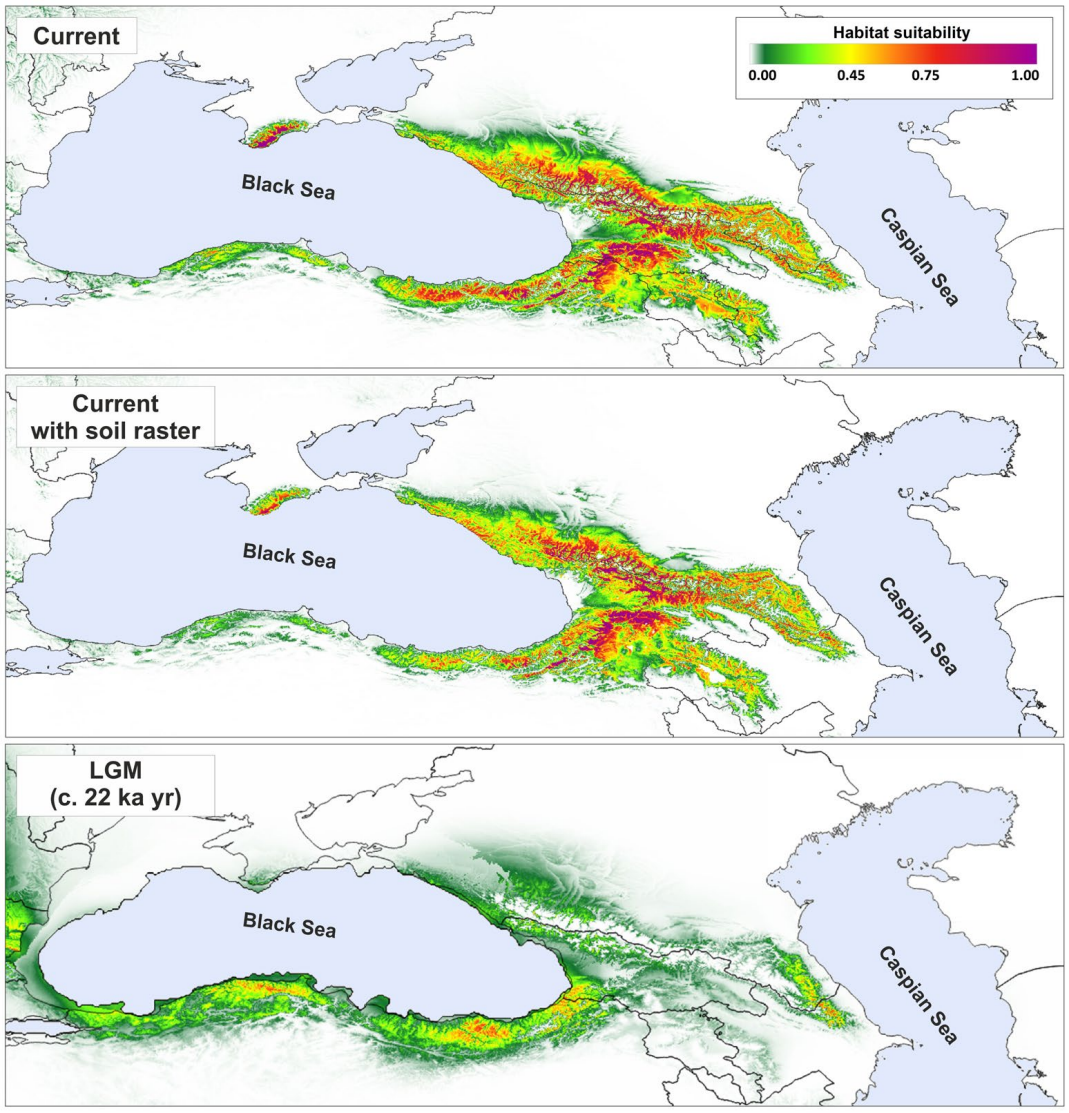
Theoretical range of Scots pine in the Caucasus and Anatolia in different periods estimated with MaxEnt based on raster data from CHELSA database. Current—current theoretical range; Current with soil raster—current theoretical range with soil preferences; LGM—theoretical range during last glacial maximum. Figure generated with QGIS 3.16 (https://qgis.org/en/site/).

Prediction of the current theoretical range of *P. sylvestris* was very similar with and without using soil raster data (Fig. 5). The main difference was in the Pontic Mts. where the soil component for the *P. sylvestris* range led to a significantly reduced occurrence rate from very high (75%) to moderate (up to 45%). Habitat suitability was also partially reduced in the eastern part of the range covering the eastern Greater Caucasus (Dagestan area) (Fig. 5)*.* The model showed the best conditions for species occurrence on the slopes of the Greater Caucasus and in the western part of the Lesser Caucasus (suitability > 0.75).

During LGM, the theoretical range of Scots pine was much reduced, and the suitability of the South Caucasus was particularly low (Fig. 5). Three major distributional centres were predicted: (1) the western part of the Pontic Mts; (2)the eastern part of the Pontic Mts. together with the Adjara located between the current territory of Georgia and Turkey; (3) the north-eastern part of the Caucasus, which currently spans the territory of southern Dagestan and northern Azerbaijan (Fig. 5). The Greater Caucasus likely did not offer suitable areas for the species presence during LGM, except for the areas mentioned above in 3). The suitability in the Greater Caucasus reached only *ca.* 30%.

**Figure 6:**
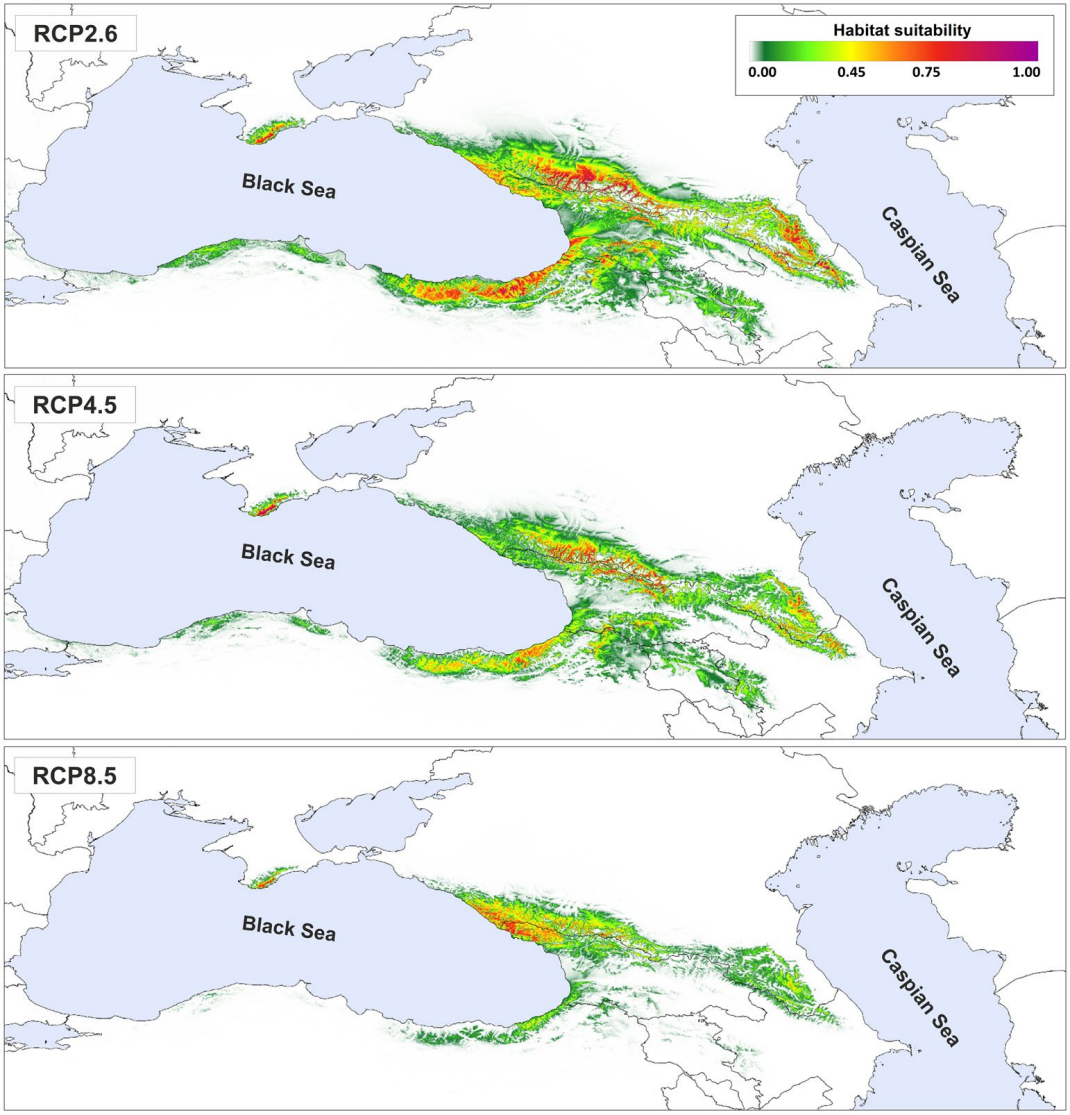
Predicted future range of Scots pine in the Caucasus and Anatolia according to different scenarios. RCP2.6—future range according to scenario RCP2.6; RCP4.5—future range according to scenario RCP4.5; RCP8.5—future range according to scenario RCP8.5. Figure generated with QGIS 3.16 (https://qgis.org/en/site/).

In the future, the suitable habitats for Scots pine are projected to decrease and most of the current populations in West Anatolia and the Lesser Caucasus are located within the area predicted to be completely unsuitable for the species (Fig. 6). A noticeable general northward shift in species distribution is also anticipated. Moreover, the model predicts that the average altitude in the theoretical range is likely to increase from the current 1409 m a.s.l. to 1790 m a.s.l. in RCP 8.5. According to this most pessimistic scenario, only Abkhazia and the adjacent part of Russia remain as a stable climatic refugia in the future; the area with high suitability (> 60%) will decrease from 81,925 km^2^ (current theoretical range) to 4922 km^2^ which is just ca. 6% of its current theoretical area (Fig. 6). Interestingly, the mountainous part of southern Crimea and Dagestan–northern Azerbaijan territories seems to offer suitable habitats continuously under the future climate.

**Figure 7:**
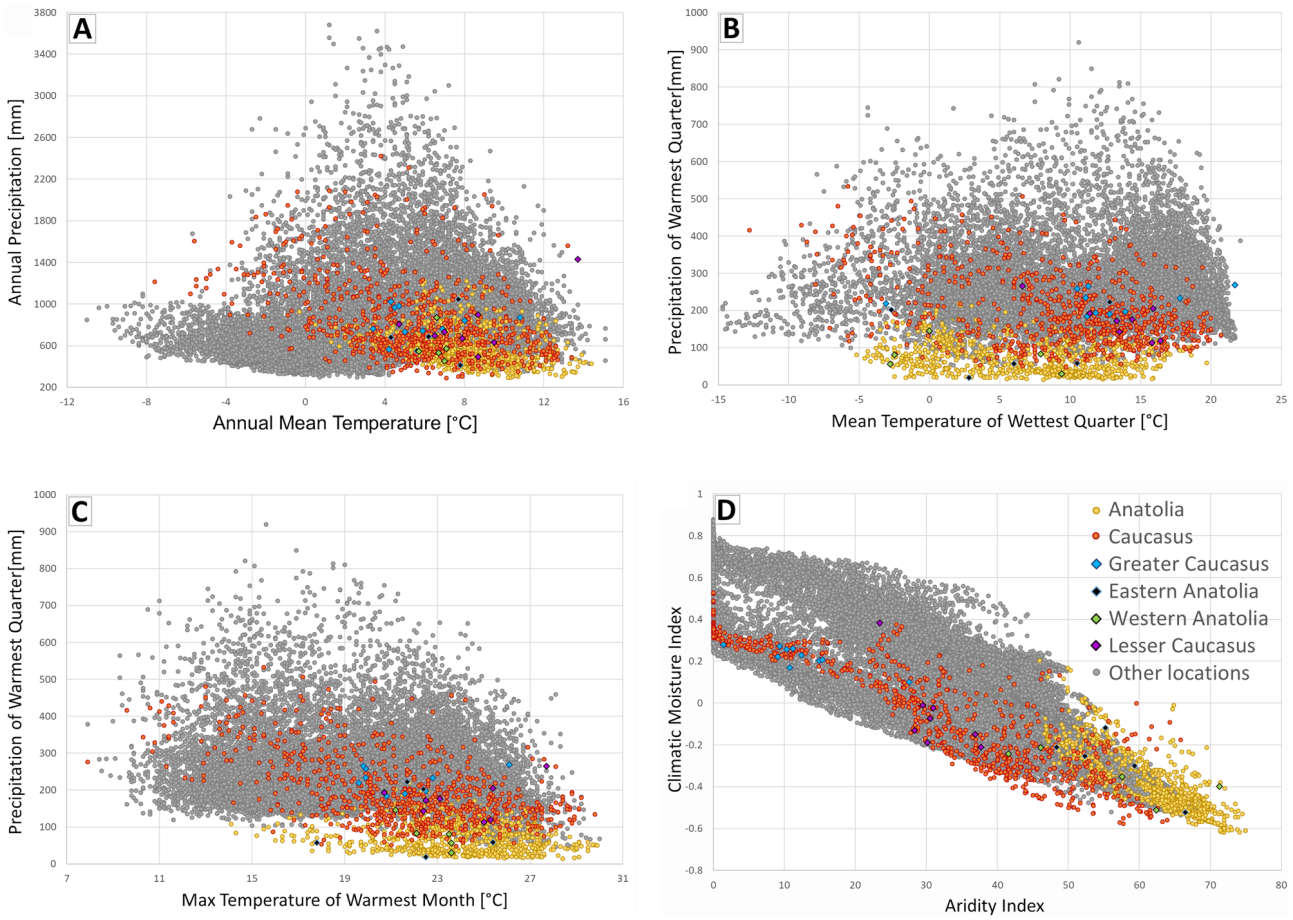
Ecoplots diagrams presenting wide-range ecological requirements of Scots pine based on the climatic variables gained from CHELSA and the ENVIREM databases: (A)—Annual Mean temperature *vs.* Annuals Precipitation; (B)—Meat temperature of Wettest Quarter *vs.* Precipitation of Warmest Quarter; (C)—Max. Temperature of Warmest Month *vs.* Precipitation of Warmest Quarter; (D)—Aridity Index *vs.* Climatic Moisture Index. The species' occurrence points were obtained from the chorological database^58^. In total, 87,834 location points we used, including those from this study. Yellow dots represent the Anatolian stands (795) and red dots refer to the Caucasian stands (1384),grey dots represent all remaining stands within the natural range of the species. Studied populations of Scots pine are denoted with additional colours (legend on the top right corner of figure D).

The ecoplots obtained for Scots pine from the South Caucasus showed that the species generally occurs in a broad bioclimatic spectrum which partly overlaps with other parts of the natural range (Fig. 7). The Anatolian sites occupied the most marginal conditions for *P. sylvestris* in terms of precipitation, temperature and aridity, and the South Caucasus sites were located immediately next to them. Both regions were very similar with respect to potential evapotranspiration which was among the highest noted in the species natural range. Among the studied populations, those from the Greater and the Lesser Caucasus differed markedly with respect to precipitation. This was especially true in Fig. 7D showing climatic moisture index *vs*. aridity index. Populations from the Lesser Caucasus generally were located in areas with lower precipitation and higher temperatures.

Generally, the populations from Anatolia occupied the most extreme climatic conditions in terms of the moisture-related traits, such as mean precipitation of warmest quarter, aridity index and potential evapotranspiration (Supplementary file 1, Table S8). Anatolia is also the area with the lowest annual precipitation, a characteristic partly shared with locations on the Crimea. In contrast, climatic conditions of the Caucasus are closer to other areas in Europe than to Anatolia, The highest precipitation in the Caucasus occurs during the winter months (similar to the Crimea, Pyrenees and Scotland) while the summer months are the wettest in the remaining geographical domains. Populations in the Caucasus get a similar level of rainfall during the warmest quarter to the Balkans, Central Europe or Scandinavia. The aridity index in the Caucasus is the lowest among investigated range domains, except for Scotland, and is far lower than that in the neighbouring Anatolia.

**Figure 8:**
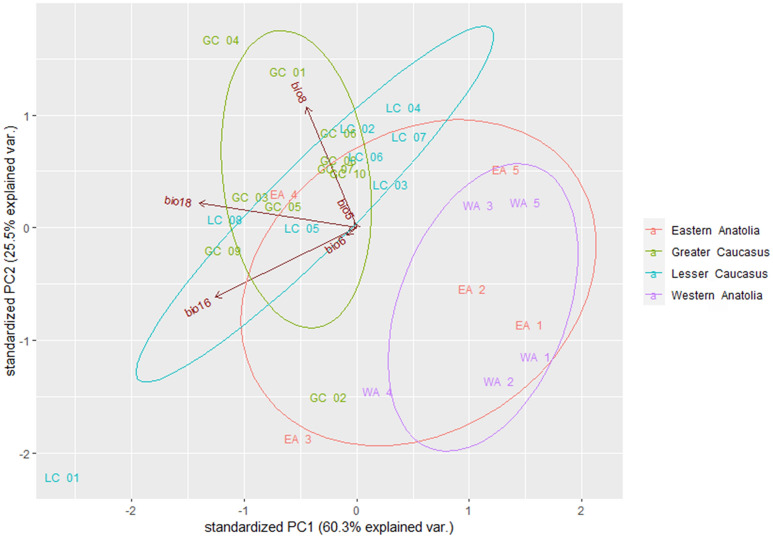
Principal component analysis (PCA) showing the climatic profiles of 28 studied populations of Scots pine based on the five bioclimatic variables that had the greatest impact on the distribution of the species in the Caucasus ecoregion (*bio5*—Max Temperature of Warmest Month, *bio6*—Min Temperature of Coldest Month, *bio8*—Mean Temperature of Wettest Quarter, *bio16*—Precipitation of Wettest Quarter and *bio18*—Precipitation of Warmest Quarter). The population acronyms are given in Table 1.

The PCA analysis performed for 28 studied populations based on the five most relevant bioclimatic variables indicated significant differentiation among them (Fig. 8). Generally, the Anatolian and Caucasian stands occupied different climatic spaces. However, closer inspection of the populations from the Caucasus mountains revealed that those from the Greater Caucasus grow under very distinct climatic conditions even though they are geographically proximate (*e.g.* GC_01 and GC_4 *vs*. GC_02). There is less pronounced climatic variability within the Lesser Caucasus—here the most extreme position is occupied by population LC_01 located in the most humid area in Adjara.

## Discussion

### Glacial refugia and Holocene divergence of Scots pine in the Caucasus

Our study revealed that the South Caucasian populations of Scots pine share the mitotype *d* dominating in Anatolia^23^, and thus populations from both regions may be of common origin. Combining genetic and geological data, it is very likely that the ancient colonization of the South Caucasus by Scots pine proceeded from the west via the Asia Minor Peninsula^23,60^.

The high allelic diversity and numerous private alleles found in the Anatolian populations strongly support the hypothesis that the Pontic Mts. served as the LGM refugium for Scots pine, an idea that has been previously presented for the species^20,21^. Notably, the results of the niche modelling for Scots pine suggest the pervasive distribution of the species during LGM in two sub-areas: 1) West Pontic Mts., and 2) East Pontic Mts. along with the Adjara (Fig. 5). Recently, the Pontic Mts. were defined as the probable refugial area for *Fagus orientalis*^61^. Palaeoenvironmental, pollen and genetic data concurrently point to the Pontic Mts.-Adjara as part of the wider refugial area defined for the Caucasian biota in areas surrounding the coasts of the Black Sea^20,62–64^. Generally, the reconstructed vegetation in the Caucasus from the Early Holocene, beyond the Colchis Lowland, indicates a domination of treeless landscape and delayed expansion of forests in comparison to Europe due to spring dryness that occurred in that period^65,66^. At some locations, a high level of *Pinus* pollen dated to the Late Pleistocene is reported and could indicate a local presence of the species outside the major Pontic Mts.-Adjara refugium^64^.

Assuming the generation time for Scots pine to be 20–25 years, the divergence within the Anatolian-South Caucasian genetic pool might have taken place at the transition between the Pleistocene and Holocene, between *ca*. 11,220 (95% CI 2940–34,600) and 14,025 (95% CI 3675–43,250), years BP, respectively. This suggests that the present genetic structure of *P. sylvestris* in the Caucasus ecoregion is a result of recent postglacial history rather than vicariance in isolated refugia as it is in other European tree species^67,68^. Recently, the Holocene fragmentation was demonstrated as the significant factor shaping the spatial genetic structure in *P. nigra* in Europe^69^. The pollen records from the Caucasus suggest that the Early Holocene was climatically adverse to Scots pine and to other conifers, leading to a reduction in their abundance in the region^64^. In this period the upward migration of Scots pine probably occurred in response to warming noted in the Caucasus. This likely contributed to further fragmentation and genetic differentiation due to reduced gene flow in a complex mountainous landscape. Additionally, Scots pine is less competitive in comparison to other conifers growing in the Caucasus which could also reduce the overall probability of establishment and increased fragmentation. Pioneer and light-demanding Scots pine is outcompeted by shade-tolerant *Abies nordmanniana* and *Picea orientalis*, and pushed onto rocky and open sites of mostly southern slopes^70^. Those populations that survived during the Early Holocene in upper altitudes were likely the local foci of the much wider expansion of the species noted in the Late Holocene^64^. Similar to other conifers, the species reached its maximum abundance just *ca.* 4000 years ago thanks to the cooling of the climate (cooler winters), natural fires and, partly, human activity that promoted conifers^62^.

The demographic analysis dated the split between both Pontic genetic pools (West–East) relatively recently, *ca*. 3700–4600 years BP. The high aridity of the region 6000–3000 years BP, with a few extremely dry episodes lasting a few hundred years each, could be a possible factor in range fragmentation and further isolation^71^. Also, this genetic pattern of differentiation might be driven by the impact of local adaptation since the West Anatolian populations are very different from the East Anatolian in terms of climatic conditions. Orsini et al.^72^ argue that local adaptation may profoundly shape the pattern of neutral genetic differentiation.

The evolutionary history of Scots pine we have tried to reconstruct in this paper is based mostly on the demographic analysis performed in DIYABC which has some constraints. The first relates to the markers used in demographic estimations. The mutational properties of microsatellites are mostly unknown for particular species and the mutation rates almost certainly do not reflect those of the genome as a whole. This may bias the final dating results. Additionally, estimation of the divergence time is expressed as the number of generations. Routinely, this parameter is subsequently converted into calendar years. Thanks to this, it is possible to make a direct reference to the geological event or time period that likely induced the process. However, the critical issue is the proper delimitation of the effective generation time. In the case of organisms with non-overlapping generations there is more certainty than in long-lived trees. In trees, researchers use different surrogates for generation time such as the age at which a species starts reproduction or total life span, the first being the most frequently used. This may be a source of apparent inconsistencies among different studies because it may lead to a different reconstruction of the evolutionary history of the species. One possible solution may be comparing the most probable evolutionary scenario of long-lived organisms to one reconstructed for a co-occurring organism (parasite or mutualist) which has clearly distinguishable generations. Recently, Goczał et al.^73^ demonstrated the convergence in the evolutionary history of the herbivorous beetle and its host tree, *Picea abies*. Such an approach would be also welcomed for other trees, including Scots pine in the Caucasus.

### Genetic diversity and differentiation

The Caucasian range of Scots pine has a marginal and relic character. In the past, such populations were undervalued due to their genetic impoverishment and unpredictable demographic trends^7^. However, our study has demonstrated that, on average, the allelic and gene diversity of Scots pine populations in the South Caucasus and Anatolia is comparable to the core populations in the boreal forest^25^ and to populations on the southern margins of the European range^74–76^. Indeed, the Caucasian-Anatolian populations are even more genetically variable than populations from Central and Northern Europe^77^, but less than in isolated Alpine populations^78^. Consequently, the pattern of genetic diversity revealed for populations of Scots pine in the South Caucasus and the Asia Minor *vs*. populations in the core range is not consistent with the assumptions of the central-marginal hypothesis^79,80^ and the rear-edge concept provides a better explanation^81^. Furthermore, there is a growing agreement that the rear-edge and relic tree populations may display high regional genetic diversity, mostly due to their complex history^68,82–84^.

However, despite overall high genetic diversity, high inbreeding levels have been detected in some Anatolian populations. Additionally, gene flow was shown to be severely reduced among populations in the West Pontic Mts., and half of the Anatolian populations experienced a bottleneck. Although the exact time of the bottlenecks have not been inferred, it could be a genetic echo of the Mid-Holocene arid episodes that could contribute to the differentiation between Anatolian lineages. The impact of more recent fragmentation by humans on diversity loss cannot be ruled out. The low level of differentiation might even favour this hypothesis, because the effects of habitat fragmentation and population size reduction in trees can be buffered by their longevity^85,86^.

The overall genetic differentiation among populations was medium, though higher than that typically noted in the European range^25,77^. The latest expansion in the Late Holocene might have contributed to an intensification of gene flow. Similar levels of differentiation to the Anatolian-Caucasian stands were reported for mountainous populations in the Carpathians and Apennines^27,78^. However, we noted that the level of regional-level *F*_*ST*_ varied considerably and was highest in the Greater Caucasus (*F*_*ST*_ = 0.074). Though located within the same mountain range, the Greater Caucasus populations are subjected to different local climatic conditions, which have been suggested in our ecological analysis. The western Greater Caucasus receives much more precipitation but summer is relatively cool while the eastern part is warmer and with significantly less precipitation^87^. Even geographically close populations within Svaneti range (GC_02 *vs*. GC_01 and GC_03) displayed very distinct climatic profiles that could induce adaptation to specific habitats. In this case, isolation by adaptation might explain the pattern of differentiation^72^. In summary, the exceptional genetic differentiation in the Greater Caucasus appears to have a solid ecological background.

The clustering of Anatolian populations of Scots pine separates western stands from the eastern stands. A similar spatial pattern of genetic structure was presented for *Castanea sativa*^88^ and *Abies nordmanniana*^89^. In a study by Wójkiewicz at.^25^ that included limited number of stands from Turkey, with western and eastern populations grouped together, only the results of PCoA suggested some distinctiveness between them. More comprehensive sampling in the region would allow us to gain insight into this subtle genetic structure.

**Figure 9:**
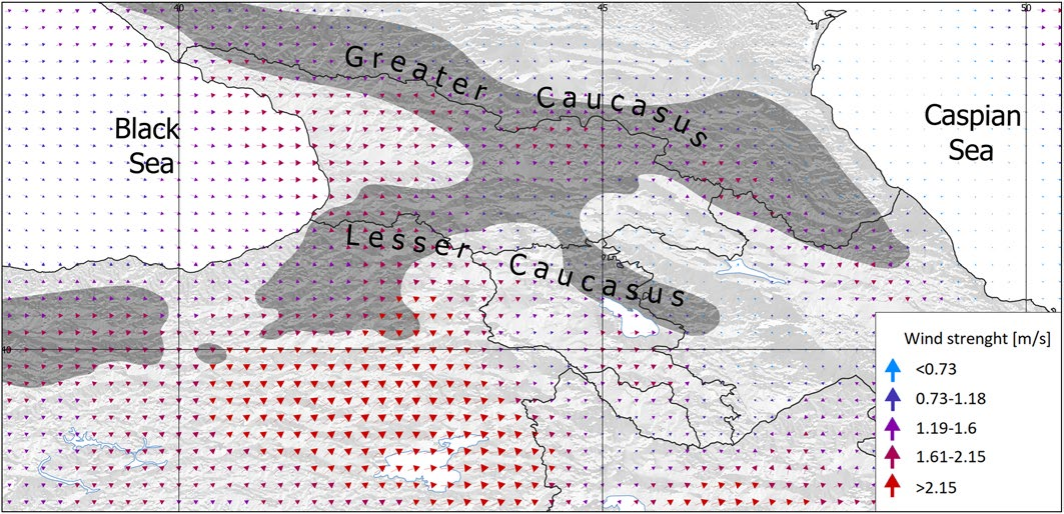
Wind pattern in the Caucasus ecoregion in May (the flowering period of Scots pine) in the period 2000–2012. The arrows indicate the direction of the wind currents while the colour and size indicate the strength. W*indscape* R package^93^ was used for data on wind currents, available in Climate System Forecast Reanalysis with a resolution of *c. 35* km^50^. Figure generated with QGIS 3.16 (https://qgis.org/en/site/).

The genetic distinctiveness of Scots pine populations from the Greater and the Lesser Caucasus reported here is also known for other species, despite the relatively close distance between both mountain ranges, which is less than 100 km^89–92^. The main barrier for gene flow retrieved with the EEMS analysis encompasses mostly the western part of the South Caucasus (Fig. 3). Low landscape connectivity due to lack of suitable habitats and topographic complexity might explain the pattern of genetic differentiation. However, an important factor might also be wind currents, accordingly to the recent concept of Isolation-by-Wind (IBW) . One study demonstrated that the wind pattern and strength both profoundly shape the landscape patterns of genetic differentiation. In Georgia, the major wind currents from the Black Sea run in the corridor between the Greater and the Lesser Caucasus toward the east but they are weakened by the Likhi range which links these ranges in Central Georgia (Figs. 1 and 9). This mountain range also has a profound impact on the climate of the South Caucasus by regulating precipitation in the region^87^. Consequently, up to the Likhi range we may assume a more linear flow of genes among populations located along both mountain ranges but not between them. After crossing the Likhi range, the influence of winds from the Black Sea is disrupted by currents from the Caspian Sea and the Armenian Highland that may affect the gene flow intensity and directionality, as stated in the IBW model. The genetic similarity of the two populations located in the Pshav-Khevsureti and Alazani ranges in the Greater Caucasus (GC_09 and 10), two others from the Lesser Caucasus (LC_05 and LC_06) and, to some extent, the Gombori range (GR), is puzzling considering their geographic locations. However, the issue becomes clearer if the landscape-level pattern of wind currents is considered (Fig. 9).

### Ecological difference of the Caucasian and Anatolian populations of Scots pine

Currently, populations from the margins of a species’ environmental niche attract evolutionists and forest geneticists because of their possible (or unique) adaptive potential^10^. The wide distributional range of Scots pine facilitates the development of local/regional adaptations to different environmental variables that have been documented using provenance trials^94^ and genomic data^95^.

Populations in the South Caucasus showed great variability in terms of the selected climatic factors. Our ecoplots showed that the studied populations overlap significantly with those representing the wide European range. Thus, being geographically marginal, the South Caucasian range of *P. sylvestris* cannot be defined as purely ecologically marginal, although some populations occupied an extreme position (*e.g*. LC_01 with the highest annual precipitation). However, the Caucasus does not seem to be typically marginal but rather an isolated geographic domain that allowed large effective populations to survive. Genomic adjustments were likely necessary for species during its postglacial range formation in a complex net of Caucasian habitats and changing climate during the Holocene. Comparing the current and the LGM distribution of Scots pine in the Pontic Mts., an eastward range shift is evident in the Holocene. Currently, the West Pontic Mts. region receives lower rainfall during spring and summer compared to the East. Consequently, the north-eastern part of the Pontic Mts. possesses a Eurosiberian character while the north-western part is more Mediterranean, suggesting that the species was able to follow the changing environmental conditions in the past. Along with a range reorganization, Scots pine likely produced local adaptations which allowed survival under changed climate. Currently, precipitation and temperature are predicted to be the two main drivers of local adaptation in a changing climate^96^.

The Asia Minor Peninsula represents the most arid and warmest place within the Scots pine niche. These are genuinely ecologically marginal populations. Unfortunately, these populations, frequently of low-density, are dispersed in a landscape profoundly changed by human activity. The excess of inbreeding detected in Anatolian populations might be the first signal of the adverse genetic processes that take place in small populations in harsh conditions.

## Conservation remarks

The future projection for the Caucasus region clearly show that the Scots pine range may be profoundly reduced or will no longer support the species. The South Caucasian-Pontic genetic pool of Scots pine is thus at high risk of extinction that would deprive us of the valuable and adaptive genetic diversity. We stress that SDMs does not consider the adaptive potential stored in the populations. Including this variable in the modelling may deliver more detailed information about the species response to climate change^97^. The wide autecological variability of the South Caucasian populations of Scots pine and the climatic marginality of the Anatolian range are likely signs of the local adaptations that should be preserved. Intriguingly, the spatial scale of adaptation in the Caucasus was very small compared to the main European range. Such a local, or microgeographic, adaptation suggests that the selective pressure on the species genetic pool was probably strong.

The evolution of new adaptations in trees is based on existing rather than de novo genetic variation^98^ and acts as polygenic selection^99^. In this context, the relatively high genetic diversity in the South Caucasian and Pontic populations deserves special conservation measures. The current health state of Turkish forests shows some negative impact of ongoing climate change. For example, the level of defoliation of trees in most Turkish forests reached 25% during 2010 to 2018, and climatic factors, particularly drought stress, are described as the main drivers (foresteurope.org). According to EUFORGEN there are 21 Genetic Conservation Units (GCU) designed for Scots pine in Turkey. Our sampling covers the areas in where the GCUs are located so the obtained results are the most up-to-date source of population genetic structure trends. Scots pine forests in Georgia are protected within the network of national parks and nature reserves that covers *ca*. 10% of the country’s forest area^70^. Based on our results we are convinced that populations in the Svaneti range (western Greater Caucasus) should be immediately incorporated into a national network of protected areas since they represent a very unique genetic pool. Ideally, the result of our investigation could serve as the basis for setting the Conservation Units (CUs) for *P. sylvestris* var. *hamata* in Georgia.

## Supplementary Information

Supplementary Information 1.

Supplementary Information 2.
