# Supplementary material for: The evolutionary heritage and ecological uniqueness of Scots pine in the Caucasus ecoregion is at risk of climate changes
Source: Sci Rep. 2021 Nov 24;11:22845. doi: 10.1038/s41598-021-02098-1 (PMC8613269; doi:10.1038/s41598-021-02098-1)
Supplement: Supplementary file 1 — Supplementary Information 1. [file 41598_2021_2098_MOESM1_ESM.docx]

Supplementary information 1 for:

**The evolutionary heritage and ecological uniqueness of Scots pine in the Caucasus ecoregion is at risk of climate changes**

Monika Dering^1,2*^, Marlena Baranowska^2^, Berika Beridze^1^, Igor J. Chybicki^3^, Irina Danelia^4^, Grzegorz Iszkuło^1,5^, Piotr Kosiński^1,2^, Giorgi Kvartskhva^4^, Peter Thomas^5^, Grzegorz Rączka^2^, Dominik Tomaszewski^1^, Łukasz Walas^1^, Katarzyna Sękiewicz^1^

*corresponding Author: [mdering@man.poznan.pl](mailto:mdering@man.poznan.pl), monikadering@gmail.com

^1^ Institute of Dendrology, Polish Academy of Sciences, Parkowa 5, 62-025 Kórnik, Poland

^2^ Poznań University of Life Sciences, Wojska Polskiego 71a, 60-625 Poznań, Poland

^3^ Department of Genetics, Faculty of Biological Sciences, Kazimierz Wielki University, Powstańców Wielkopolskich 10, 85-090, Bydgoszcz, Poland

^4^ Agricultural Science and Bio-system Engineering, Georgian Technical University, Guramishvili Str. 17, 0192 Tbilisi, Georgia

^5^ Faculty of Biological Sciences, University of Zielona Góra, Prof. Z. Szafrana 1, 65-516 Zielona Góra, Poland

^6^ School of Biological Sciences, Keele University, Staffordshire, ST5 5BG, UK

**Distribution and taxonomy**

*Pinus sylvestris* var. *hamata* Steven is endemic to the Caucasus: synonyms include *P. kochiana* Klotzsch ex K.Koch., *P. sosnovskyi* Nakai*, P. hamata* (Steven) Sosn. and *P. caucasica* (Medw.) N.Busch but Farjon (2015) treats it as a subspecies of *P. sylvestris.* The taxon is apparently morphologically different from a typical Scots pine from the boreal range but detailed morphological analysis has yet to be done (Farjon 2015; Akhalkatsi et al. 2019). It is distributed in the Lesser Caucasus, the Greater Caucasus, the Pontic Mts. and the Armenian Upland. The species rarely forms dense stands. The populations are mostly isolated and small but these plant communities have a significant conservation value due to rich species diversity (Akhalkatsi and Tarkhnishvili, 2012; Akhalkatsi et al., 2019). As a pioneer and light-demanding species, in the Greater and Lesser Caucasus, it is outcompeted by shade-tolerant *Abies nordmanniana* and *Picea orientalis*, and pure stands are noted only on rocky slopes. As an admixture, Scots pine is reported from the conifer forests and beech forests of the Greater and Lesser Caucasus and in Colchis broadleaved forests (Akhalkatsi and Tarkhnishvili, 2012). Below are photos that exemplifie the character of the species occurrence in Georgia.


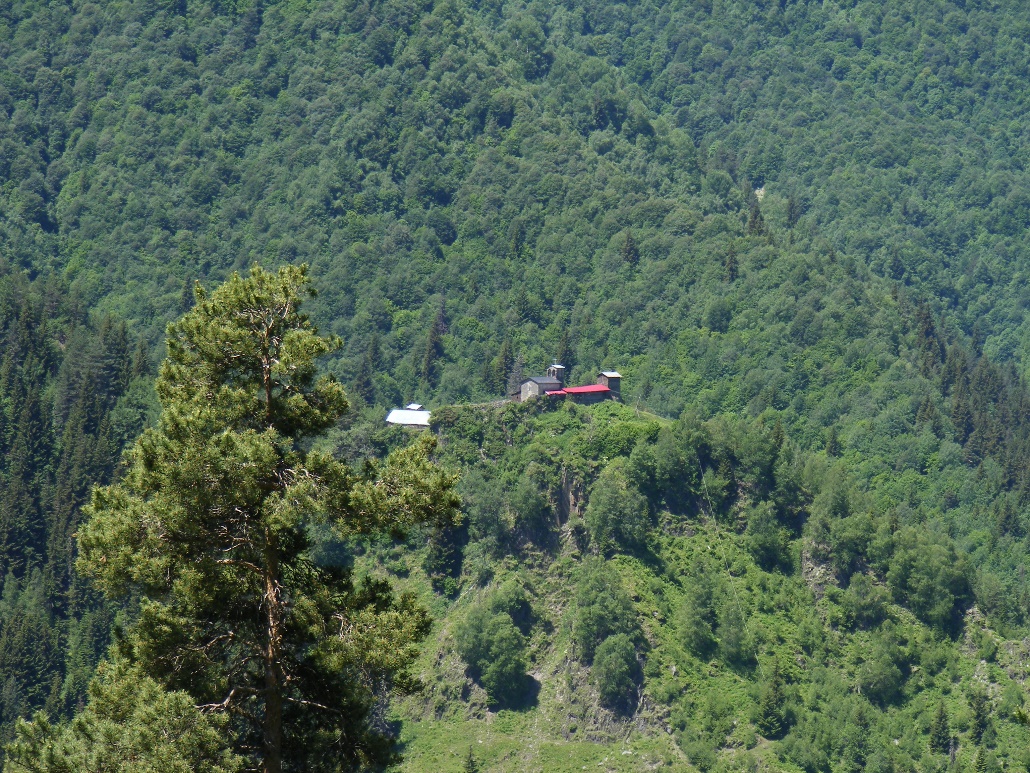


**Photo 1.** Natural stand of *Pinus sylvestris* var*. hamata* in Svaneti Region, Georgia, The Greater Caucasus (Author: G. Iszkuło).


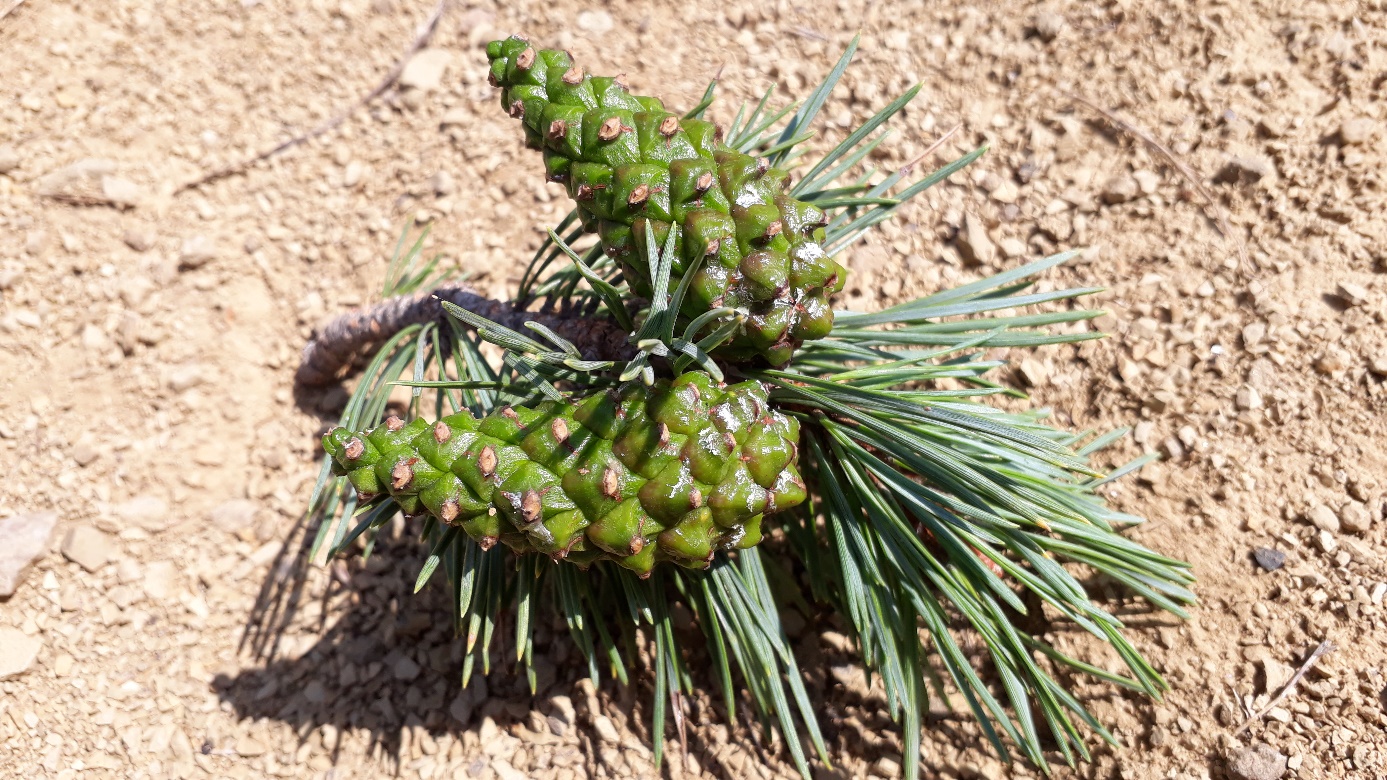


**Photo 2**. Immature cones of *Pinus sylvestris* var. *hamata* in Svaneti Region, Georgia, The Greater Caucasus (Author: G. Iszkuło).


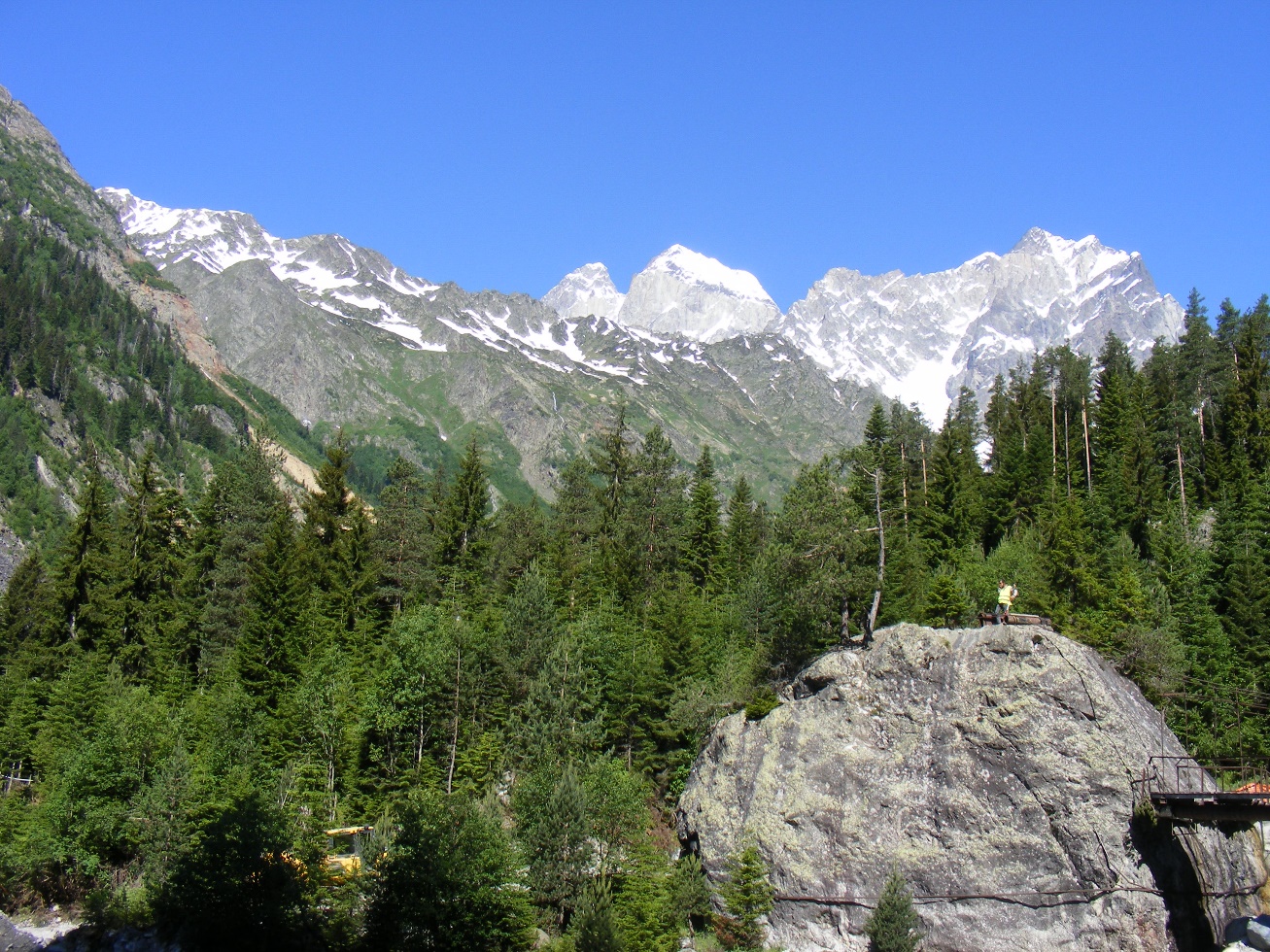


**Photo 3.** *Pinus sylvestris* var. *hamata* in mixed forests with *Abies nordmanniana* and *Picea orientalis*, Svaneti Region, near Mestia, Georgia, The Greater Caucasus (Author: G. Iszkuło).


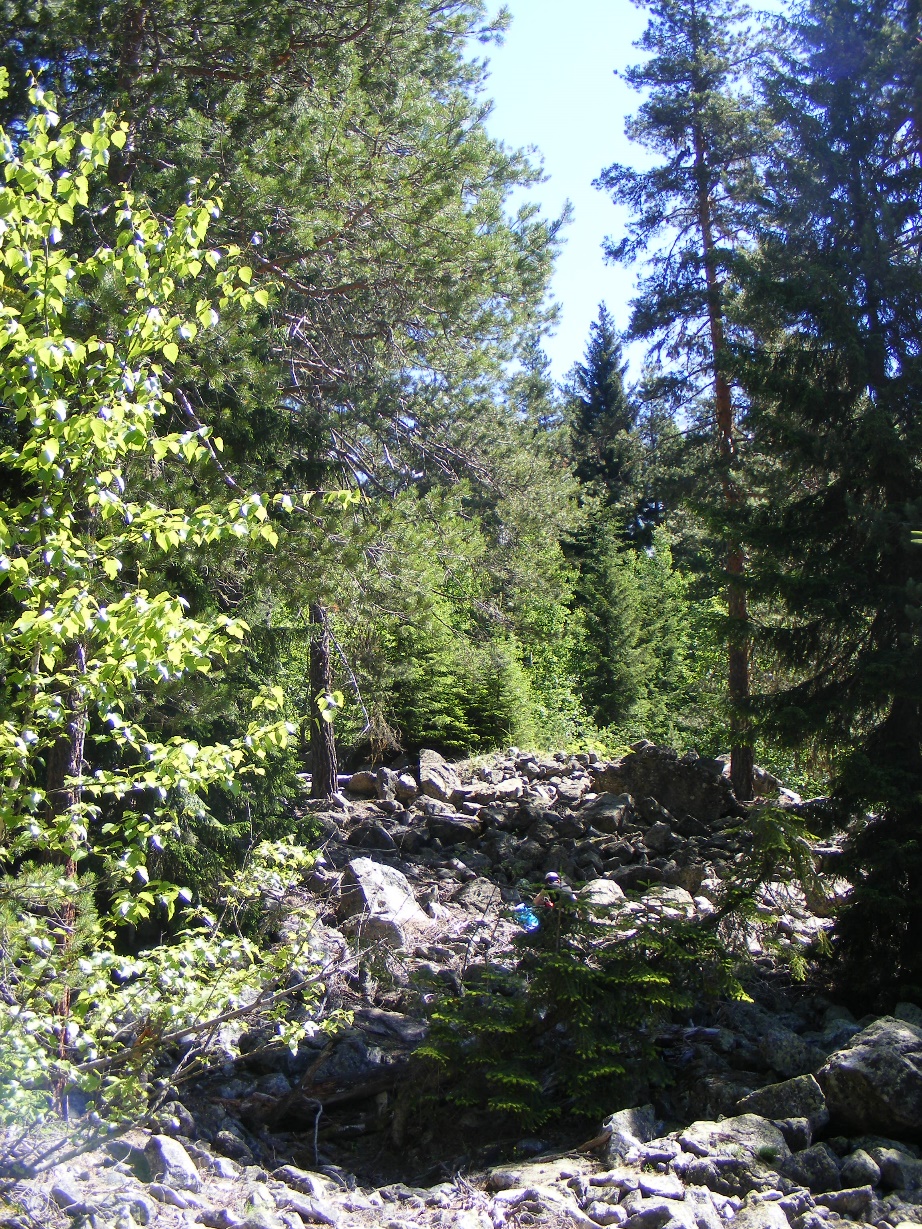


**Photo 4.** *Pinus sylvestris* var. *hamata* in mixed forests with *Abies nordmanniana* and *Picea orientalis*, Svaneti Region, Georgia, The Greater Caucasus (Author: G. Iszkuło).

**Literature**

Akhalkatsi, M., Tarkhnishvili, D. 2012. Habitats of Natura 2000 in Georgia are developed in the framework of the project of GTZ. Tbilisi.

Akhalkatsi, M., Arabuli, G., Asanidze, Z., Goloshvili, T. & Togonidze, N. 2019. Pine Forest on Tree-Line Ecotone in the Mountain Kazbegi in the Georgia (South Caucasus). *Agricultural Research* 7.

Akhalkatsi, M. 2019. Plant species in Natura 2000 habitats in Georgia. Tbilisi.

Farjon, A. 2017. Handbook of the World's Conifers (2 vols.): Revised and Updated Edition, p.790. Brill Academic Publishers.

**Table S1.** Locations of natural populations of Scots pine studied in this study (GC – The Greater Caucasus, LC – The Lesser Caucasus, WA – West Anatolia, EA – East Anatolia)

| **Population ID** | **Locality** | **N** | **Latitude** | **Longitude** | **Elevation (m a.s.l)** |
| --- | --- | --- | --- | --- | --- |
|  |  |  |  |  |  |
| GC_01 | Georgia. Svaneti Range | 33 | 43.052 | 42.434 | 1117 |
| GC_02 | Georgia. Svaneti Range | 30 | 43.111 | 42.744 | 1678 |
| GC_03 | Georgia. Svaneti Range | 31 | 43.027 | 42.718 | 1814 |
| GC_04 | Georgia. Racha Range | 30 | 42.503 | 43.143 | 798 |
| GC_05 | Georgia. Shoda-Kedela Range | 18 | 42.775 | 43.513 | 1678 |
| GC_06 | Georgia. Shoda-Kedela Range | 30 | 42.751 | 43.537 | 1307 |
| GC_07 | Georgia. Shoda-Kedela Range | 11 | 42.692 | 43.691 | 1646 |
| GC_08 | Georgia. Shoda-Kedela Range | 19 | 42.700 | 43.679 | 1523 |
| GC_09 | Georgia. Pshav-Khevsureti Range | 33 | 42.603 | 45.103 | 1803 |
| GC_10 | Georgia. Alazani Range | 32 | 42.364 | 45.625 | 1899 |
| GR | Georgia. Gombori Range | 31 | 41.759 | 45.381 | 1160 |
| LC_01 | Georgia. Adjara-Imereti Range | 30 | 41.598 | 41.922 | 527 |
| LC_02 | Georgia. Adjara-Imereti Range | 30 | 41.669 | 42.636 | 1320 |
| LC_03 | Georgia. Adjara-Imereti Range | 30 | 41.786 | 42.846 | 1413 |
| LC_04 | Georgia. Trialeti Range | 31 | 41.810 | 43.452 | 1005 |
| LC_05 | Georgia. Trialeti Range | 32 | 41.721 | 43.495 | 1863 |
| LC_06 | Georgia. Trialeti Range | 30 | 41.827 | 43.858 | 1475 |
| LC_07 | Georgia. Trialeti Range | 31 | 41.705 | 44.345 | 1209 |
| WA_01 | Turkey. West Anatolia | 25 | 39.960 | 31.110 | 1619 |
| WA_02 | Turkey. West Anatolia | 31 | 40.640 | 32.410 | 1419 |
| WA_03 | Turkey. West Anatolia | 30 | 41.120 | 34.060 | 1583 |
| WA_04 | Turkey. West Anatolia | 30 | 41.640 | 34.830 | 1228 |
| WA_05 | Turkey. West Anatolia | 30 | 39.960 | 36.520 | 1579 |
| EA_01 | Turkey. East Anatolia | 31 | 39.870 | 39.050 | 2010 |
| EA_02 | Turkey. East Anatolia | 28 | 40.610 | 39.410 | 1980 |
| EA_03 | Turkey. East Anatolia | 30 | 41.150 | 41.760 | 1663 |
| EA_04 | Turkey. East Anatolia | 34 | 41.230 | 42.430 | 1700 |
| EA_05 | Turkey. East Anatolia | 24 | 40.180 | 42.630 | 1850 |
| ***Average across all populations*** | |  |  |  |  |

N- number of individuals analysed

**Table S2**. Details of multiplex PCRs applied in the work.

| Mutiplex | Loci/concentration | Anneling temperature/time |
| --- | --- | --- |
| Muliti I | Psyl57 ((0.075µM)  Psyl25 (0.025µM)  Psyl17 (0.015µM)  Psyl36 (0.035µM) | 55˚C/60s |
| Muliti II | Psyl44 (0.035µM)  Psyl42 (0.020µM)  Psyl19 (0.010µM)  Psyl2 (0.025µM)  Psyl16 (0.050µM) | 55˚C/60s |
| Muliti III | SPAC7.14 (0.075µM) SPAC11.4 (0.075µM) SPAC11.8 (0.025µM)  SPAC12.5 (0.050µM) | 56˚C/60s |

PCR multiplex reactions in a total volume of 10µl with ca. 50 ng DNA, 10×Silver Hot Buffer, 2.5mM MgCl2, 0.2mM dNTPs, 0.5U Sliver Hot Polymerase (Syngen, Poland). The PCR thermal protocol was an initial denaturation at 95˚C for 15min, and subsequently 35 cycles of: denaturation at 95˚C/30s, annealing temperature (as in Table S2), elongation at 72˚C/60s and final elongation at 72˚C/10 min.


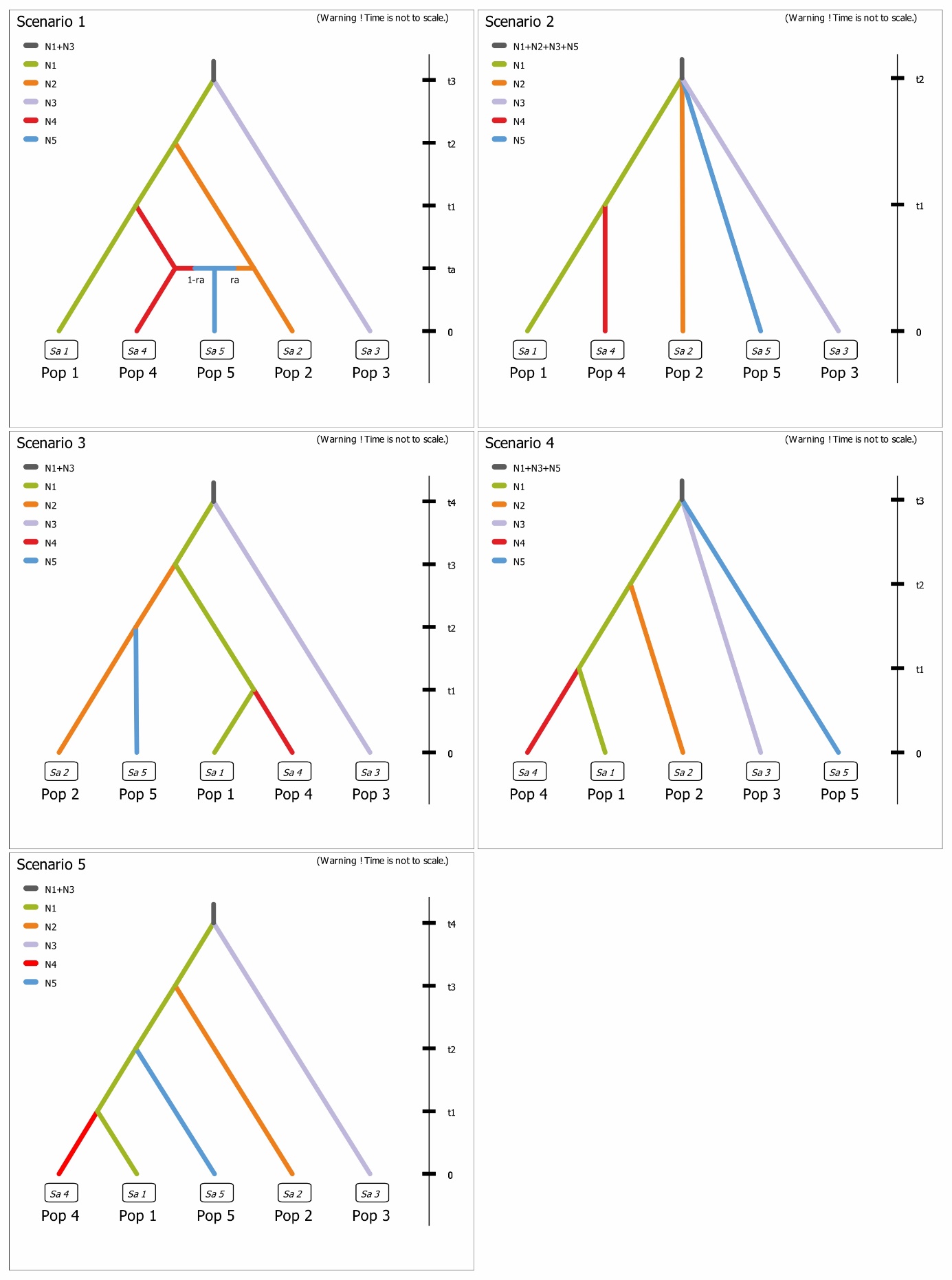


**Figure S1.** Demographic scenarios tested with ABC method performed in DIYABC for Scots pine in the Caucasus and Anatolia based on nuclear microsatellites. Pop1 – West Anatolia, Pop2 - West Greater Caucasus, Pop3 – Central Caucasus, Pop4 – East Anatolia, Pop5 – West Lesser Caucasus. Scenario 2 was indicated as the most likely.

**Table S3.** Genetic and historical parameters used in demographic analysis conducted with ABC method performed in DIYABC based on nSSRs

| **Parameters** | **Distribution** | **Min-Max** | **Mean** | **Shape** |
| --- | --- | --- | --- | --- |
| **Genetic parameters** |  |  |  |  |
| **Set 1** (psyl_12, psyl_17, psyl_19, psyl_25, psyl_36, psyl_42, psyl_44, psyl_57, SPAC11.4 | | | | |
| Mean mutation rate | Log uniform | 1.10^-7^-1.10^-4^ |  |  |
| Individual mutation rate | Gamma | 1.10^-9^-1.10^-3^ | Mean mutation rate | 2 |
| Mean coefficient P | Uniform | 0.1-9 |  |  |
| Individual locus coefficient P | Gamma | 0.1-10 | Mean coefficient P | 2 |
| **Set 2** (SPAC7.14, SPAC11.8, SPAC12.5, psyl_16) | | | | |
| Mean mutation rate | Log uniform | 1.10^-4^-1.10^-2^ |  |  |
| Individual mutation rate | Gamma | 1.10^-5^-1.10^-2^ | Mean mutation rate | 2 |
| Mean coefficient P | Uniform | 0.1-5 |  |  |
| Individual locus coefficient P | Gamma | 0.1-10 | Mean coefficient P | 2 |
| **Historical parameters** | | | | |
| West Anatolia |  | 100-32000 |  |  |
| West Greater Caucasus |  | 100-18000 |  |  |
| Central Caucasus |  | 10-10000 |  |  |
| East Anatolia |  | 10-10000 |  |  |
| West Lesser Caucasus |  | 10-10000 |  |  |
| *Ta* |  | 10-1000 |  |  |
| *Ra* |  | 0.001-0.999 |  |  |
| *t1* |  | 10-2000 |  |  |
| *t2* |  | 10-3000 |  |  |
| *t3* |  | 50-8000 |  |  |
| *t4* |  | 100-10000 |  |  |
| Conditions | t1>ta, t2>ta, t2>t1, t3>t2, t4>t3 | | | |


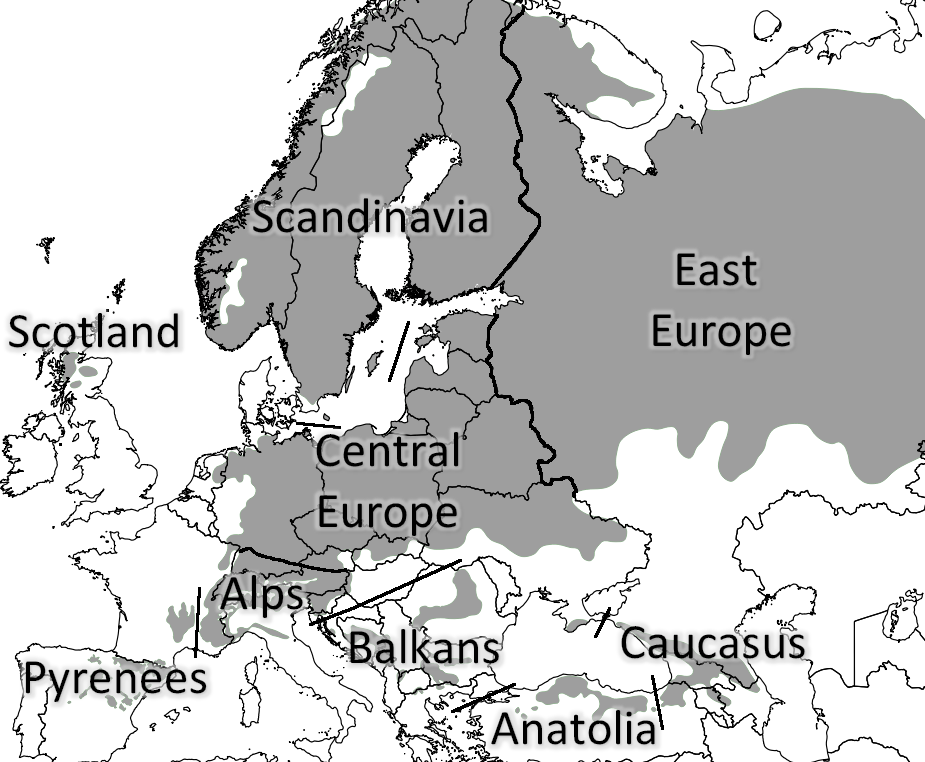


**Fig. S2.** The geographic domains in the natural range of Scots pine analysed in terms of the

ecological differences. Map generated with QGIS 3.16 Figure generated with QGIS 3.16 (https://qgis.org/en/site/).


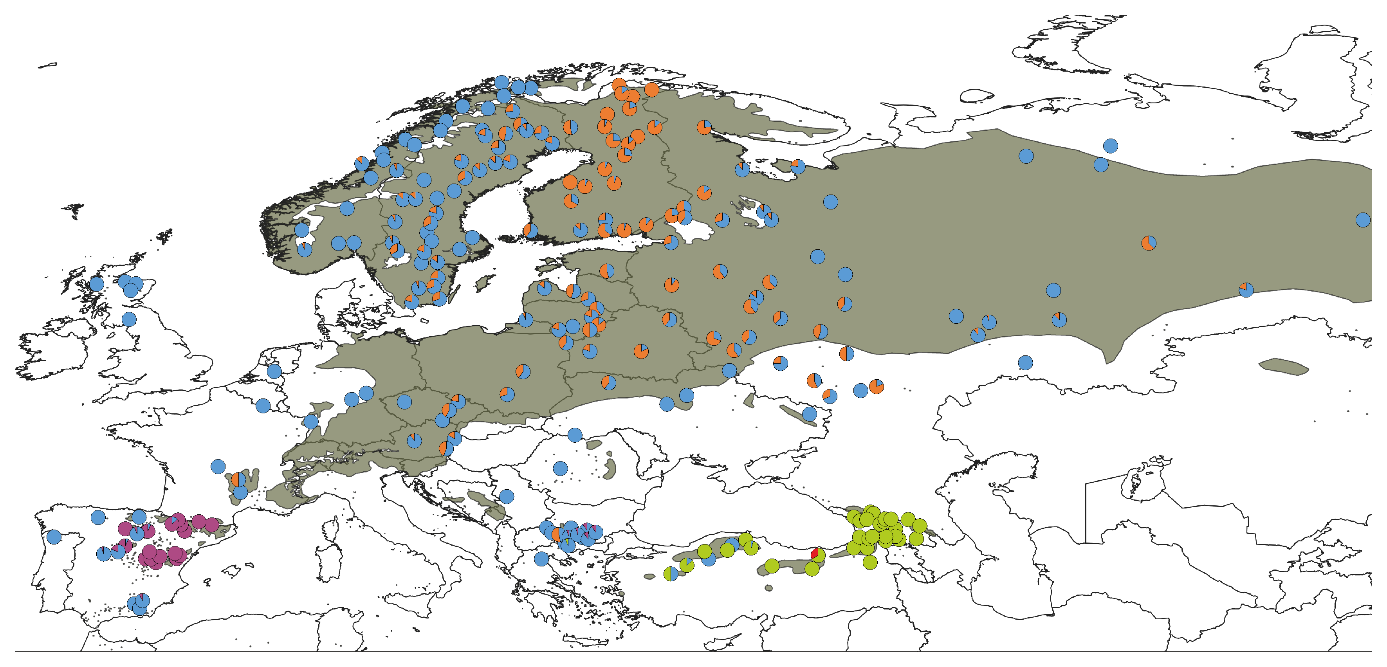


**Fig. S3.** The distribution of mitotype of mtDNA in Scots pine across its natural range. Pie charts denote the frequency of mitotype *nad7* and *nad1*(Naydenov et al. 2007) detected in each of the populations. Blue-mitotype *a,* orange – mitotype *b*, violet – mitotype *c*, green – mitotype *d*. The data obtained in this work that covers only the Caucasus and a few samples in Anatolia were compiled with results presented in Dering et al. 2017. Populations analysed in this work (Georgia) all contained mitotype *d*. Map generated with QGIS 3.16 (https://qgis.org/en/site/).

**Fig. S4**. The optimal K number detected with procedure of Puechmaille (2016) applied in STRUCTURE SELECTOR.

**Table S4.**  The coefficient of the membership (Q) to each of clusters inferred with STRUCTURE for 28 populations of Scots pine in the Anatolia and the Caucasus

| **Population ID** | **Cluster I** | **Cluster II** | **Cluster III** | **Cluster IV** | **Cluster V** |
| --- | --- | --- | --- | --- | --- |
|  |  |  |  |  |  |
| GC_01 | 0.027 | 0.799 | 0.007 | 0.135 | 0.032 |
| GC_02 | 0.061 | 0.800 | 0.014 | 0.079 | 0.046 |
| GC_03 | 0.017 | 0.908 | 0.009 | 0.028 | 0.039 |
| GC_04 | 0.030 | 0.713 | 0.024 | 0.162 | 0.071 |
| GC_05 | 0.041 | 0.768 | 0.033 | 0.116 | 0.042 |
| GC_06 | 0.032 | 0.806 | 0.046 | 0.070 | 0.046 |
| GC_07 | 0.025 | 0.843 | 0.027 | 0.040 | 0.065 |
| GC_08 | 0.028 | 0.647 | 0.096 | 0.175 | 0.058 |
| GC_09 | 0.062 | 0.019 | 0.504 | 0.183 | 0.233 |
| GC_10 | 0.112 | 0.040 | 0.456 | 0.120 | 0.272 |
| GR | 0.096 | 0.041 | 0.314 | 0.088 | 0.461 |
| LC_01 | 0.078 | 0.060 | 0.010 | 0.497 | 0.355 |
| LC_02 | 0.070 | 0.071 | 0.043 | 0.726 | 0.091 |
| LC_03 | 0.104 | 0.038 | 0.032 | 0.705 | 0.121 |
| LC_04 | 0.059 | 0.113 | 0.064 | 0.721 | 0.044 |
| LC_05 | 0.092 | 0.063 | 0.384 | 0.369 | 0.092 |
| LC_06 | 0.032 | 0.028 | 0.711 | 0.062 | 0.115 |
| LC_07 | 0.080 | 0.023 | 0.075 | 0.352 | 0.470 |
| WA_01 | 0.843 | 0.028 | 0.023 | 0.037 | 0.069 |
| WA_02 | 0.729 | 0.026 | 0.010 | 0.047 | 0.188 |
| WA_03 | 0.737 | 0.020 | 0.038 | 0.110 | 0.094 |
| WA_04 | 0.589 | 0.037 | 0.192 | 0.086 | 0.095 |
| WA_05 | 0.530 | 0.058 | 0.130 | 0.075 | 0.208 |
| EA_01 | 0.441 | 0.047 | 0.173 | 0.053 | 0.286 |
| EA_02 | 0.213 | 0.040 | 0.202 | 0.073 | 0.471 |
| EA_03 | 0.212 | 0.026 | 0.233 | 0.063 | 0.465 |
| EA_04 | 0.046 | 0.056 | 0.255 | 0.161 | 0.481 |
| EA_05 | 0.153 | 0.038 | 0.233 | 0.089 | 0.488 |


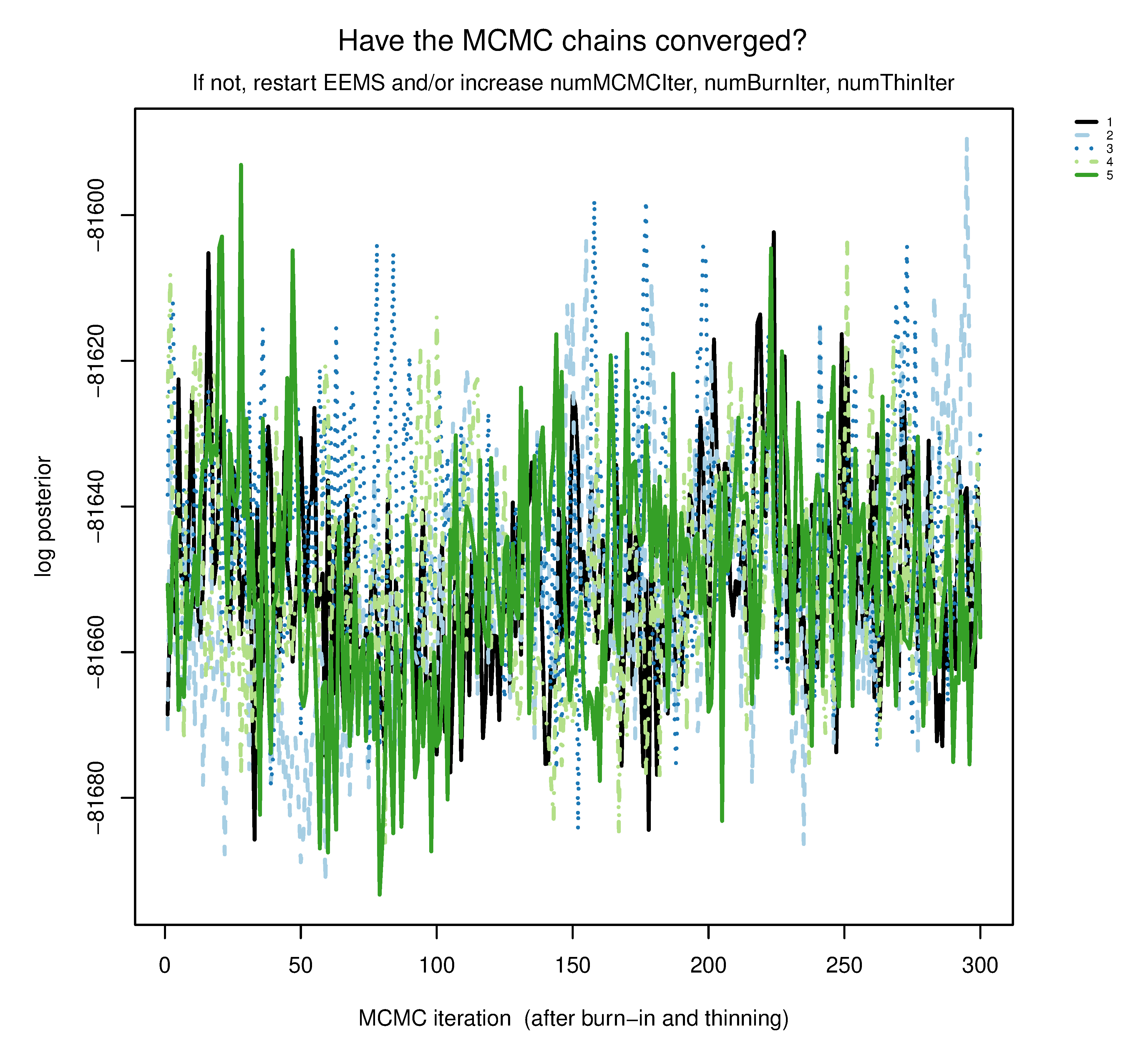


**Fig. S5.** The convergence of five chains used in EEMS analysis showing the good performance of the analysis. Each line (blue, green, dotted, dashed and dashed-dotted) represents each of the five chains used in the analysis.

**Table S5.** The M-ratio test for bottleneck under the two-phase model (TPM) estimated for studied populations of Scots pine; population abbreviations as in Table 1

| **Population**  **ID** | **MR** | **MReq** | **P-value** |
| --- | --- | --- | --- |
| **GC_01** | **0.678** | **0.777** | **0.0052** |
| GC_02 | 0.731 | 0.777 | 0.1899 |
| **GC_03** | **0.698** | **0.784** | **0.0482** |
| GC_04 | 0.724 | 0.764 | 0.2130 |
| GC_05 | 0.680 | 0.747 | 0.1172 |
| GC_06 | 0.708 | 0.790 | 0.0559 |
| GC_07 | 0.673 | 0.717 | 0.1721 |
| GC_08 | 0.726 | 0.758 | 0.2141 |
| GC_09 | 0.770 | 0.784 | 0.3132 |
| GC_10 | 0.717 | 0.782 | 0.1520 |
| GR | 0.725 | 0.778 | 0.1037 |
| LC_01 | 0.673 | 0.769 | 0.1390 |
| LC_02 | 0.714 | 0.782 | 0.1347 |
| **LC_03** | **0.692** | **0.772** | **0.0321** |
| LC_04 | 0.703 | 0.782 | 0.0656 |
| LC_05 | 0.692 | 0.767 | 0.1713 |
| LC_06 | 0.726 | 0.781 | 0.0653 |
| **LC_07** | **0.618** | **0.775** | **0.0107** |
| **WA_01** | **0.601** | **0.767** | **0.0135** |
| **WA_02** | **0.607** | **0.750** | **0.0133** |
| WA_03 | 0.692 | 0.772 | 0.0644 |
| WA_04 | 0.694 | 0.767 | 0.1230 |
| **WA_05** | **0.607** | **0.750** | **0.0133** |
| **EA_01** | **0.637** | **0.769** | **0.0342** |
| EA_02 | 0.666 | 0.772 | 0.0738 |
| EA_03 | 0.703 | 0.781 | 0.1169 |
| **EA_04** | **0.609** | **0.774** | **0.0062** |
| EA_05 | 0.661 | 0.753 | 0.0890 |

**Abbreviations:** MR – the mean observed M-ratio; MReq – the M-ratio generated under mutation-drift equilibrium and P-value – probability of significant test for the deficiency in M-ratio based on Wilcoxon signed-ranks test. Significant values of M-ratio and P-value are in bold

**Details of the stages of the analysis of divergence time performed for *Pinus sylvestris* var. *hamata* in DIYABC software (based on nuclear microsatellites; nSSRs)**


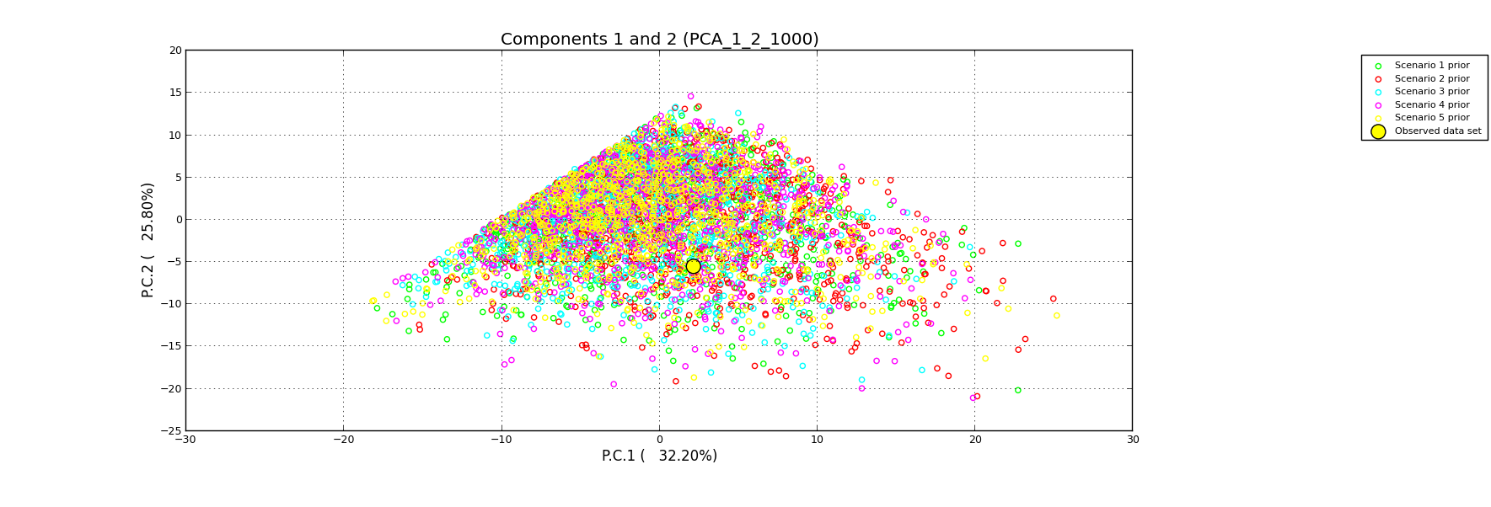


**Figure S6.** Principal component analysis performed in DIYABC to evaluate the simulated data sets to test for the scenarios of divergence done with ABC procedure. Each color dot represents simulated data for each of the five scenarios tested while big yellow dot represent the observed data set used in the analysis (nSSRs genotypes).

**Table S6.** Values of each summary statistics for all demographic scenarios tested with ABC method performed in DIYABC and the proportion of simulated data sets which have a value below the observed one.

| Statistics | Value | Scenario 1 | Scenario 2 | Scenario 3 | Scenario 4 | Scenario 5 |
| --- | --- | --- | --- | --- | --- | --- |
| NAL_1_1 | (8.0000) | 0.9096 | 0.8680 | 0.9119 | 0.8979 | 0.9115 |
| NAL_1_2 | (7.7778) | 0.9502 (*) | 0.9099 | 0.9569 (*) | 0.9418 | 0.9562 (*) |
| NAL_1_3 | (6.3333) | 0.9672 (*) | 0.9221 | 0.9741 (*) | 0.9636 (*) | 0.9739 (*) |
| NAL_1_4 | (6.5556) | 0.9458 | 0.8984 | 0.9359 | 0.9249 | 0.9364 |
| NAL_1_5 | (6.0000) | 0.9213 | 0.9094 | 0.9614 (*) | 0.9542* | 0.9475 |
| HET_1_1 | (0.4895) | 0.8370 | 0.7550 | 0.8412 | 0.8130 | 0.8408 |
| HET_1_2 | (0.5126) | 0.8881 | 0.8109 | 0.9047 | 0.8687 | 0.9032 |
| HET_1_3 | (0.5022) | 0.9288 | 0.8428 | 0.9430 | 0.9167 | 0.9424 |
| HET_1_4 | (0.4788) | 0.8740 | 0.7918 | 0.8686 | 0.8444 | 0.8680 |
| HET_1_5 | (0.4239) | 0.8187 | 0.7591 | 0.8771 | 0.8593 | 0.8488 |
| VAR_1_1 | (3.4985) | 0.5125 | 0.4183 | 0.5202 | 0.4839 | 0.5205 |
| VAR_1_2 | (2.6725) | 0.5046 | 0.4031 | 0.5294 | 0.4734 | 0.5274 |
| VAR_1_3 | (3.2378) | 0.6057 | 0.4674 | 0.6340 | 0.5768 | 0.6332 |
| VAR_1_4 | (3.1218) | 0.5393 | 0.4363 | 0.5379 | 0.5033 | 0.5378 |
| VAR_1_5 | (2.8030) | 0.5093 | 0.4489 | 0.5786 | 0.5569 | 0.5505 |
| MGW_1_1 | (0.4966) | 0.2602 | 0.2628 | 0.2563 | 0.2657 | 0.2571 |
| MGW_1_2 | (0.7254) | 0.6420 | 0.6851 | 0.6267 | 0.6605 | 0.6281 |
| MGW_1_3 | (0.7308) | 0.6036 | 0.6932 | 0.5868 | 0.6232 | 0.5858 |
| MGW_1_4 | (0.6743) | 0.5798 | 0.6322 | 0.5777 | 0.6000 | 0.5768 |
| MGW_1_5 | (0.5294) | 0.4062 | 0.4433 | 0.3928 | 0.4000 | 0.3981 |
| N2P_1_1&2 | (10.0000) | 0.9218 | 0.8786 | 0.9166 | 0.9125 | 0.9164 |
| N2P_1_1&3 | (8.5556) | 0.8891 | 0.8497 | 0.8899 | 0.8768 | 0.8885 |
| N2P_1_1&4 | (8.7778) | 0.9071 | 0.8674 | 0.9116 | 0.8975 | 0.9109 |
| N2P_1_1&5 | (8.5556) | 0.8957 | 0.8496 | 0.8948 | 0.8771 | 0.9011 |
| N2P_1_2&3 | (8.4444) | 0.9220 | 0.8801 | 0.9274 | 0.9111 | 0.9258 |
| N2P_1_2&4 | (8.7778) | 0.9308 | 0.8796 | 0.9221 | 0.9163 | 0.9214 |
| N2P_1_2&5 | (8.4444) | 0.9325 | 0.8804 | 0.9428 | 0.9113 | 0.9248 |
| N2P_1_3&4 | (7.4444) | 0.9115 | 0.8613 | 0.9039 | 0.8913 | 0.9039 |
| N2P_1_3&5 | (6.8889) | 0.8846 | 0.8555 | 0.9130 | 0.9020 | 0.9029 |
| N2P_1_4&5 | (7.5556) | 0.9249 | 0.8672 | 0.9131 | 0.8954 | 0.9182 |
| H2P_1_1&2 | (0.5239) | 0.8643 | 0.7819 | 0.8631 | 0.8433 | 0.8622 |
| H2P_1_1&3 | (0.5164) | 0.8506 | 0.7790 | 0.8497 | 0.8270 | 0.8487 |
| H2P_1_1&4 | (0.4892) | 0.8433 | 0.7589 | 0.8468 | 0.8186 | 0.8463 |
| H2P_1_1&5 | (0.4683) | 0.8163 | 0.7215 | 0.8160 | 0.7787 | 0.8240 |
| H2P_1_2&3 | (0.5504) | 0.8885 | 0.8256 | 0.8935 | 0.8685 | 0.8921 |
| H2P_1_2&4 | (0.5161) | 0.8693 | 0.7850 | 0.8662 | 0.8467 | 0.8650 |
| H2P_1_2&5 | (0.5007) | 0.8675 | 0.7733 | 0.8875 | 0.8254 | 0.8606 |
| H2P_1_3&4 | (0.5007) | 0.8551 | 0.7803 | 0.8504 | 0.8281 | 0.8498 |
| H2P_1_3&5 | (0.4924) | 0.8489 | 0.7803 | 0.8632 | 0.8431 | 0.8558 |
| H2P_1_4&5 | (0.4627) | 0.8399 | 0.7343 | 0.8262 | 0.7897 | 0.8342 |
| V2P_1_1&2 | (3.1585) | 0.4879 | 0.3845 | 0.4840 | 0.4588 | 0.4845 |
| V2P_1_1&3 | (3.4475) | 0.4868 | 0.4008 | 0.4858 | 0.4532 | 0.4856 |
| V2P_1_1&4 | (3.3371) | 0.5043 | 0.4077 | 0.5130 | 0.4763 | 0.5129 |
| V2P_1_1&5 | (3.2423) | 0.4931 | 0.3918 | 0.4904 | 0.4443 | 0.5032 |
| V2P_1_2&3 | (3.0327) | 0.4760 | 0.3883 | 0.4793 | 0.4415 | 0.4792 |
| V2P_1_2&4 | (2.9707) | 0.4858 | 0.3812 | 0.4812 | 0.4555 | 0.4811 |
| V2P_1_2&5 | (2.8450) | 0.4907 | 0.3790 | 0.5192 | 0.4330 | 0.4801 |
| V2P_1_3&4 | (3.2442) | 0.4901 | 0.4010 | 0.4867 | 0.4538 | 0.4861 |
| V2P_1_3&5 | (3.1240) | 0.4832 | 0.4012 | 0.4947 | 0.4650 | 0.4886 |
| V2P_1_4&5 | (3.0338) | 0.5037 | 0.3913 | 0.4903 | 0.4442 | 0.5023 |
| FST_1_1&2 | (0.0801) | 0.5182 | 0.4858 | 0.2538 | 0.5390 | 0.2540 |
| FST_1_1&3 | (0.0817) | 0.1210 | 0.3338 | 0.0767 | 0.1113 | 0.0767 |
| FST_1_1&4 | (0.0197) | 0.1543 | 0.2445 | 0.3009 | 0.2800 | 0.2993 |
| FST_1_1&5 | (0.0239) | 0.1484 | 0.0611 | 0.0524 | 0.0362 (*) | 0.1134 |
| FST_1_2&3 | (0.1474) | 0.2358 | 0.5231 | 0.1391 | 0.2236 | 0.1418 |
| FST_1_2&4 | (0.0711) | 0.2830 | 0.2731 | 0.1568 | 0.3368 | 0.1559 |
| FST_1_2&5 | (0.0853) | 0.6226 | 0.2742 | 0.3829 | 0.0975 | 0.1672 |
| FST_1_3&4 | (0.0472) | 0.0576 | 0.1013 | 0.0491 (*) | 0.0499 (*) | 0.0495 (*) |
| FST_1_3&5 | (0.1081) | 0.1379 | 0.2674 | 0.0804 | 0.0970 | 0.0840 |
| FST_1_4&5 | (0.0273) | 0.2214 | 0.0522 | 0.0548 | 0.0374 (*) | 0.1028 |
| AML_1_5&2&4 | (0.2450) | 0.2097 | 0.2229 | 0.0871 | 0.2878 | 0.4988 |
| NAL_2_1 | (22.5000) | 0.3543 | 0.3062 | 0.3623 | 0.3459 | 0.3603 |
| NAL_2_2 | (18.7500) | 0.3491 | 0.2953 | 0.3650 | 0.3401 | 0.3610 |
| NAL_2_3 | (16.0000) | 0.4284 | 0.3609 | 0.4430 | 0.4261 | 0.4442 |
| NAL_2_4 | (19.2500) | 0.4617 | 0.3992 | 0.4443 | 0.4331 | 0.4436 |
| NAL_2_5 | (16.2500) | 0.3641 | 0.3747 | 0.4415 | 0.4362 | 0.4176 |
| HET_2_1 | (0.8524) | 0.3530 | 0.2786 | 0.3581 | 0.3378 | 0.3575 |
| HET_2_2 | (0.8124) | 0.3399 | 0.2556 | 0.3621 | 0.3241 | 0.3580 |
| HET_2_3 | (0.8415) | 0.5505 | 0.4464 | 0.5716 | 0.5431 | 0.5707 |
| HET_2_4 | (0.8138) | 0.4059 | 0.3172 | 0.3856 | 0.3671 | 0.3831 |
| HET_2_5 | (0.8472) | 0.4804 | 0.4692 | 0.5722 | 0.5581 | 0.5359 |
| VAR_2_1 | (23.9109) | 0.1444 | 0.0914 | 0.1485 | 0.1302 | 0.1475 |
| VAR_2_2 | (25.0724) | 0.1719 | 0.1131 | 0.1908 | 0.1561 | 0.1875 |
| VAR_2_3 | (16.0014) | 0.1841 | 0.1076 | 0.2020 | 0.1737 | 0.2026 |
| VAR_2_4 | (21.0499) | 0.1691 | 0.1095 | 0.1645 | 0.1467 | 0.1643 |
| VAR_2_5 | (18.8240) | 0.1399 | 0.1224 | 0.1937 | 0.1908 | 0.1747 |
| MGW_2_1 | (0.6716) | 0.1033 | 0.1415 | 0.1011 | 0.1163 | 0.1013 |
| MGW_2_2 | (0.7282) | 0.2879 | 0.3273 | 0.2761 | 0.3084 | 0.2726 |
| MGW_2_3 | (0.7901) | 0.6714 | 0.7681 | 0.6501 | 0.7006 | 0.6479 |
| MGW_2_4 | (0.5580) | 0.1718 | 0.1960 | 0.1595 | 0.1712 | 0.1591 |
| MGW_2_5 | (0.7222) | 0.5063 | 0.6034 | 0.5108 | 0.5230 | 0.5172 |
| N2P_2_1&2 | (25.5000) | 0.3130 | 0.2427 | 0.3042 | 0.3051 | 0.3010 |
| N2P_2_1&3 | (23.5000) | 0.2775 | 0.2331 | 0.2761 | 0.2654 | 0.2743 |
| N2P_2_1&4 | (24.7500) | 0.3318 | 0.2798 | 0.3452 | 0.3279 | 0.3437 |
| N2P_2_1&5 | (23.5000) | 0.2973 | 0.2343 | 0.2917 | 0.2661 | 0.3066 |
| N2P_2_2&3 | (20.5000) | 0.2516 | 0.2045 | 0.2562 | 0.2404 | 0.2552 |
| N2P_2_2&4 | (22.7500) | 0.3075 | 0.2373 | 0.2969 | 0.2945 | 0.2942 |
| N2P_2_2&5 | (20.2500) | 0.2721 | 0.2007 | 0.2958 | 0.2350 | 0.2533 |
| N2P_2_3&4 | (21.2500) | 0.3085 | 0.2556 | 0.2991 | 0.2899 | 0.2998 |
| N2P_2_3&5 | (18.7500) | 0.2444 | 0.2198 | 0.2730 | 0.2640 | 0.2630 |
| N2P_2_4&5 | (21.5000) | 0.3432 | 0.2634 | 0.3194 | 0.2959 | 0.3296 |
| H2P_2_1&2 | (0.8600) | 0.3635 | 0.2663 | 0.3548 | 0.3475 | 0.3524 |
| H2P_2_1&3 | (0.8599) | 0.3581 | 0.2892 | 0.3561 | 0.3398 | 0.3546 |
| H2P_2_1&4 | (0.8427) | 0.3433 | 0.2646 | 0.3481 | 0.3256 | 0.3458 |
| H2P_2_1&5 | (0.8708) | 0.4031 | 0.3270 | 0.4056 | 0.3755 | 0.4204 |
| H2P_2_2&3 | (0.8400) | 0.3314 | 0.2589 | 0.3383 | 0.3115 | 0.3362 |
| H2P_2_2&4 | (0.8451) | 0.3639 | 0.2631 | 0.3463 | 0.3410 | 0.3438 |
| H2P_2_2&5 | (0.8338) | 0.3511 | 0.2417 | 0.3755 | 0.2934 | 0.3268 |
| H2P_2_3&4 | (0.8368) | 0.3545 | 0.2797 | 0.3398 | 0.3256 | 0.3388 |
| H2P_2_3&5 | (0.8510) | 0.3956 | 0.3486 | 0.4306 | 0.4157 | 0.4176 |
| H2P_2_4&5 | (0.8483) | 0.4291 | 0.3166 | 0.3910 | 0.3615 | 0.4060 |
| V2P_2_1&2 | (24.7890) | 0.1342 | 0.0767 | 0.1276 | 0.1198 | 0.1265 |
| V2P_2_1&3 | (20.7501) | 0.1040 | 0.0649 | 0.1012 | 0.0889 | 0.1008 |
| V2P_2_1&4 | (22.5333) | 0.1323 | 0.0805 | 0.1378 | 0.1194 | 0.1371 |
| V2P_2_1&5 | (22.0291) | 0.1249 | 0.0690 | 0.1182 | 0.0938 | 0.1284 |
| V2P_2_2&3 | (21.6710) | 0.1127 | 0.0714 | 0.1118 | 0.0966 | 0.1118 |
| V2P_2_2&4 | (23.3083) | 0.1320 | 0.0751 | 0.1243 | 0.1168 | 0.1241 |
| V2P_2_2&5 | (22.7402) | 0.1379 | 0.0752 | 0.1560 | 0.1012 | 0.1246 |
| V2P_2_3&4 | (19.0728) | 0.1038 | 0.0635 | 0.0993 | 0.0871 | 0.0995 |
| V2P_2_3&5 | (17.3785) | 0.0948 | 0.0604 | 0.0984 | 0.0880 | 0.0959 |
| V2P_2_4&5 | (20.2549) | 0.1300 | 0.0686 | 0.1155 | 0.0924 | 0.1253 |
| FST_2_1&2 | (0.0640) | 0.7128 | 0.7044 | 0.5824 | 0.7264 | 0.5851 |
| FST_2_1&3 | (0.0271) | 0.1302 | 0.2255 | 0.1076 | 0.1304 | 0.1091 |
| FST_2_1&4 | (0.0214) | 0.2712 | 0.3787 | 0.4035 | 0.3969 | 0.4055 |
| FST_2_1&5 | (0.0465) | 0.5787 | 0.4357 | 0.3183 | 0.2885 | 0.4791 |
| FST_2_2&3 | (0.0388) | 0.1774 | 0.2848 | 0.1486 | 0.1756 | 0.1503 |
| FST_2_2&4 | (0.0719) | 0.5853 | 0.5975 | 0.4989 | 0.6269 | 0.5021 |
| FST_2_2&5 | (0.0202) | 0.2779 | 0.0968 | 0.1214 | 0.0480 (*) | 0.0644 |
| FST_2_3&4 | (0.0261) | 0.0573 | 0.1154 | 0.0525 | 0.0650 | 0.0537 |
| FST_2_3&5 | (0.0143) | 0.0096 (**) | 0.0212 (*) | 0.0054 (**) | 0.0076 (**) | 0.0061 (**) |
| FST_2_4&5 | (0.0482) | 0.5466 | 0.3102 | 0.2327 | 0.2071 | 0.3538 |
| AML_2_5&2&4 | (0.7080) | 0.7270 | 0.6784 | 0.4554 | 0.6675 | 0.8253 |
| FST_2_3&5 (0.0143) | | 0.0096 (**) | 0.0212 (*) | 0.0054 (**) | 0.0076 (**) | 0.0061 (**) |
| FST_2_4&5 (0.0482) | | 0.5466 | 0.3102 | 0.2327 | 0.2071 | 0.3538 |


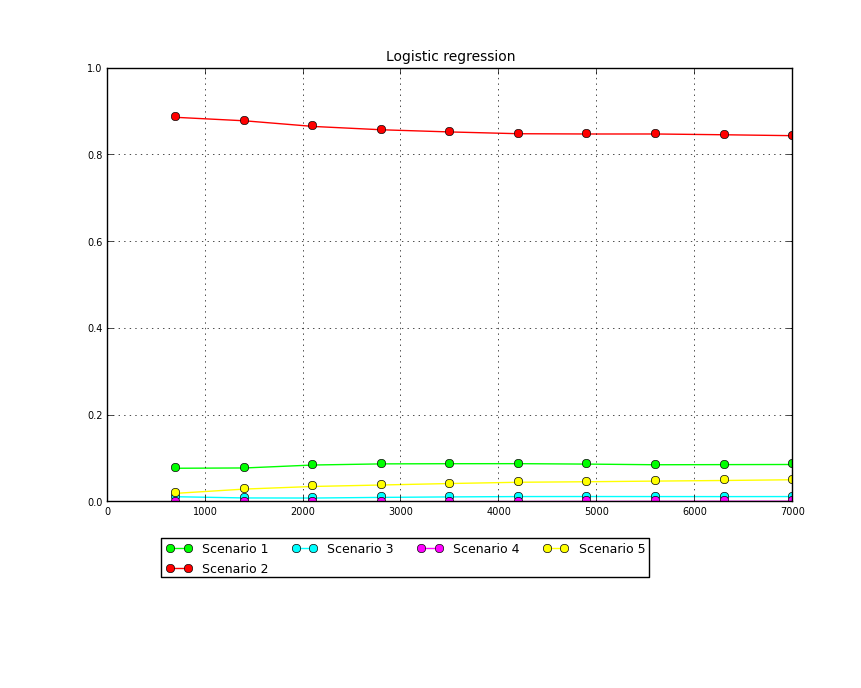


**Fig. S7.** Posterior probability obtained for five tested demographic scenarios of divergence tested with ABC method implemented in DIYABC – a logistic regression. Scenario 2 was indicated as heaving the highest posterior probability and so was indicated as the most probable one for populations of Scots pine in the Caucasus and Anatolia.

**Table S7.** Confidence in demographic scenarios tested with ABC method implemented in DIYABC for Scots pine. For each scenario, 500 pseudoobserved data sets were produced by simulations. The type I and II errors associated with Scenario 2 and other tested scenarios are presented.

| Type II Error Type I Error | | | | | | |
| --- | --- | --- | --- | --- | --- | --- |
| **Scenario 1** | | **2** | **3** | **4** | **5** |  |
| True Scenario used for simulation | | | | | |  |
| **1** | - | **0.016** | 0.006 | 0.000 | 0.001 | 0.032 |
| **2** | 0.042 | **-** | 0.027 | 0.086 | 0.047 | **0.202** |
| **3** | 0.100 | **0.058** | - | 0.001 | 0.018 | 0.186 |
| **4** | 0.010 | **0.084** | 0.038 | - | 0.022 | 0.154 |
| **5** | 0.084 | **0.022** | 0.022 | 0.004 | - | 0.088 |


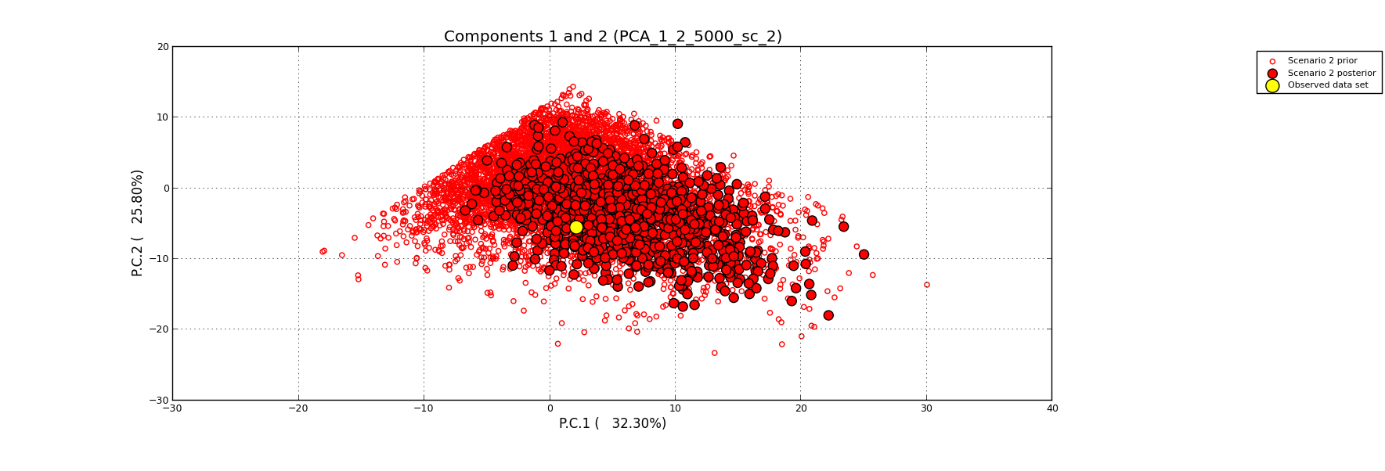

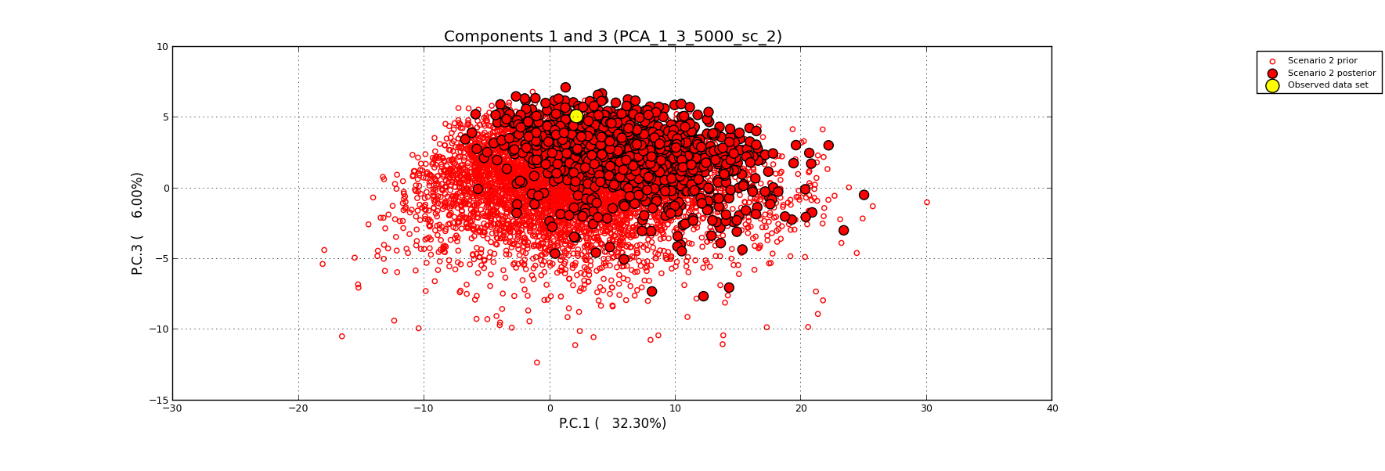

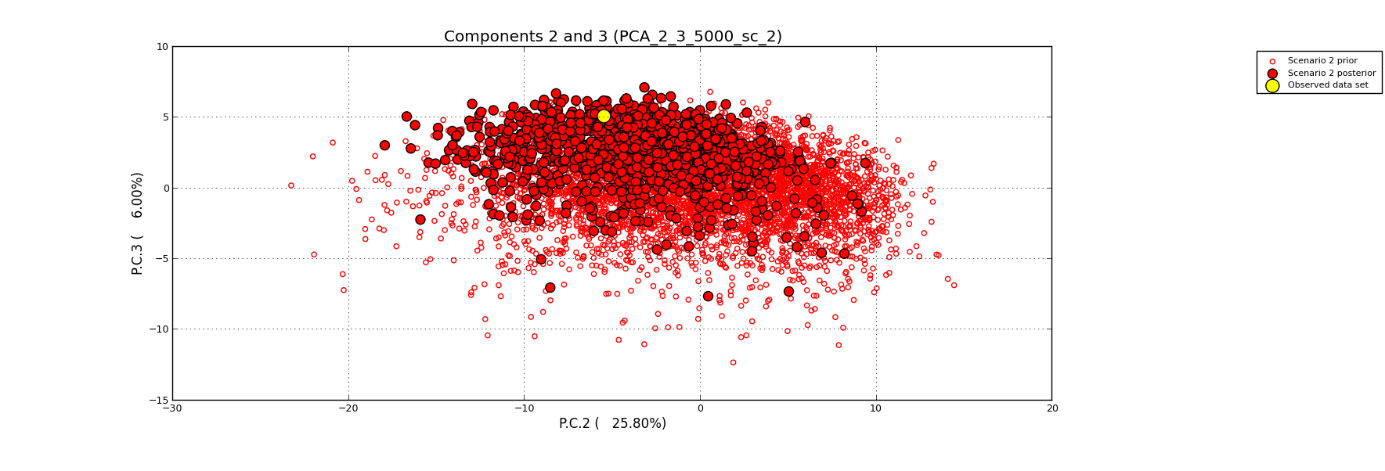


**Fig. S8.** Model checking for the best scenario of divergence indicated with ABC method. This was done for Scenario 2 that was indicated among the five tested scenarios for Scots pine in the Caucasus and Anatolia as the most likely. The purpose of this analysis is to check that at least one combination of scenarios and priors can produce simulated data (red points) sets that are close enough to the observed data set (yellow point).


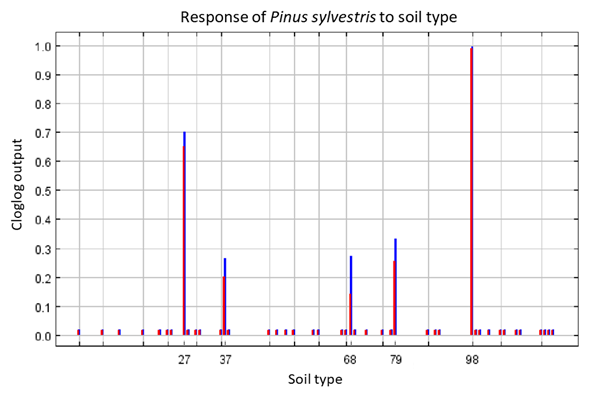


**Fig. S9.** Response of Scots pine to soil type according to MaxEnt model. The soil types bars and the respective numbers are shown on X-axis: 27 - Haplic Cambisols, 37 - Haplic Chernozems, 68 - Haplic Leptosols, 79 - Haplic Luvisols, 98 -Haplic Podzols. On Y-axis the predicted probability of presence.

| **Region** | **Annual Mean Temperature** | | | **Mean Temperature of Wettest Quarter** | | | **Annual Precipitation** | | | **Precipitation of Warmest Quarter** | | | **Aridity Index** | | | **Potential Evapotranspiration** | | |
| --- | --- | --- | --- | --- | --- | --- | --- | --- | --- | --- | --- | --- | --- | --- | --- | --- | --- | --- |
|  | Mean | | Group | Mean | | Group | Mean | | Group | Mean | | Group | Mean | | Group | Mean | std | Group |
| Alps | 7.33 | (3.29) | c | 12.13 | (5.98) | c | 1170.67 | (351.51) | b | 357.58 | (135.37) | a | 13.55 | (14.35) | h | 723.19 | (121.87) | e |
| **Anatolia** | **8.09** | **(2.41)** | **b** | **6.81** | **(5.49)** | **f** | **581.93** | **(168.55)** | **g** | **71.75** | **(40.90)** | **h** | **61.54** | **(6.88)** | **a** | **938.65** | **(74.14)** | **a** |
| Balkans | 7.41 | (2.35) | c | 12.58 | (6.38) | c | 824.75 | (256.12) | d | 225.88 | (62.82) | d | 29.74 | (14.19) | f | 792.32 | (91.76) | c |
| **Caucasus** | **4.67** | **(4.36)** | **e** | **8.94** | **(6.45)** | **e** | **882.40** | **(381.48)** | **c** | **233.89** | **(95.50)** | **c** | **26.25** | **(17.14)** | **g** | **778.80** | **(116.27)** | **d** |
| Central Europe | 7.80 | (1.15) | b | 17.53 | (2.41) | a | 647.48 | (113.56) | f | 231.75 | (37.83) | c | 37.16 | (8.79) | d | 704.74 | (63.57) | f |
| Crimea | 9.88 | (1.46) | a | 3.54 | (2.38) | g | 644.10 | (93.97) | efg | 142.86 | (22.63) | g | 55.64 | (4.64) | b | 840.41 | (38.97) | b |
| Pyrenees | 9.15 | (2.19) | a | 8.96 | (4.65) | e | 892.38 | (282.49) | c | 178.66 | (72.21) | f | 36.83 | (11.55) | d | 833.45 | (86.47) | b |
| Russia | 2.14 | (2.47) | g | 15.60 | (2.97) | b | 584.30 | (77.80) | g | 211.63 | (29.68) | e | 38.64 | (6.09) | c | 557.62 | (88.32) | g |
| Scandinavia | 2.54 | (2.51) | f | 11.53 | (5.01) | d | 704.53 | (352.95) | e | 222.32 | (58.55) | d | 31.45 | (12.62) | e | 469.44 | (72.42) | i |
| Scotland | 6.49 | (1.12) | d | 3.63 | (2.10) | g | 1608.13 | (609.21) | a | 309.85 | (98.41) | b | 9.00 | (10.23) | i | 501.42 | (23.76) | h |

**Table S8.** Mean values (SD in brackets) of bioclimatic variables among different geographical domains of Scots pine natural range
